# Tissue and cellular spatiotemporal dynamics in colon aging

**DOI:** 10.1038/s41587-025-02830-6

**Published:** 2025-10-22

**Authors:** Aidan C. Daly, Francesco Cambuli, Tarmo Äijö, Britta Lötstedt, Nemanja Despot Marjanovic, Sara Fernandez, Olena Kuksenko, Matthew Smith-Erb, Daniel Domovic, Nicholas Van Wittenberghe, Eugene Drokhlyansky, Gabriel K. Griffin, Hemali Phatnani, Richard Bonneau, Aviv Regev, Sanja Vickovic

**Affiliations:** 1 New York Genome Center, New York, NY USA; 2 Center for Computational Biology, Flatiron Institute, New York, NY USA; 3 Klarman Cell Observatory Broad Institute of MIT and Harvard, Cambridge, MA USA; 4 Science for Life Laboratory, Department of Gene Technology, KTH Royal Institute of Technology, Stockholm, Sweden; 5 Department of Biology, Massachusetts Institute of Technology, Cambridge, MA USA; 6 Department of Neurology, Columbia University Irving Medical Center, New York, NY USA; 7 Department of Pathology, Brigham and Women’s Hospital, Boston, MA USA; 8 Center for Data Science, New York University, New York, NY USA; 9 Department of Biomedical Engineering and Herbert Irving Institute for Cancer Dynamics, Columbia University, New York, NY USA; 10 Science for Life Laboratory, Department of Immunology, Genetics and Pathology, Beijer Laboratory for Gene and Neuro Research, Uppsala University, Uppsala, Sweden; 11 Present Address: Human Technopole, Milan, Italy; 12 Present Address: Genentech, San Francisco, CA USA

**Keywords:** Gene expression analysis, Bayesian inference, RNA sequencing, Ageing, Data integration

## Abstract

Tissue structure and molecular circuitry in the colon can be profoundly impacted by systemic age-related effects but many of the underlying molecular cues remain unclear. Here, we build a cellular and spatial atlas of the colon across three anatomical regions and 11 age groups, encompassing ~1,500 mouse gut tissues profiled by spatial transcriptomics and ~400,000 single nucleus RNA-sequencing profiles. We develop a computational framework, cSplotch, which learns a hierarchical Bayesian model of spatially resolved cellular expression associated with age, tissue region and sex by leveraging histological features to share information across tissue samples and data modalities. Using this model, we identify cellular and molecular gradients along the adult colonic tract and across the main crypt axis and multicellular programs associated with aging in the large intestine. Our multimodal framework for the investigation of cell and tissue organization can aid in the understanding of cellular roles in tissue-level pathology.

## Abstract (web summary)

cSplotch compares spatial transcriptomics data across ages and regions of mouse colon tissue.

## Main

A typical colon extends >12 cm in mice and >1.5 m in humans^1,2^, with considerable variance in length, thickness and folding, impacted by multiple variables, including age, sex, weight and diet. The inner lumen of the large intestine is punctuated by millions of invaginations, each harboring a colonic crypt, the key anatomic unit responsible for its continuous regeneration and differentiation^3^. Underlying the mucosal epithelium, the submucosa hosts lymphoid clusters, nerve fibers and the lymphovasculature, while the outer muscular wall enables peristaltic motility. From the cecum to the rectum, the colon carries out spatially confined regional functions, which emerge postnatally and are required for the digestion of solid food^4,5^. During aging, the decline of colonic function is accompanied by dysbiosis and excessive epithelial permeability, allowing the gut microbiota to infiltrate the lumen^6^ and causing a generalized and protracted inflammatory state^7^, as well as the emergence of common pathologies, including constipation, diverticulitis, malnutrition and colorectal cancer (CRC)^8^.

Despite the crucial importance of colon function, the cellular and molecular features associated with functional diversity across colonic regions, the crypt axis and major lifespan stages have not yet been comprehensively characterized. In recent years, single-cell and single-nucleus profiling of the mouse and human intestines^9–20^ has discovered and classified cell types and functions in the gut during development^15,20^, in adults^16,17,19^ and in aging^18,21^ but with limited spatial context. Spatial in situ profiling methods^22–33^ are poised to address this gap, through either targeted or genome-wide profiling. However, robust computational frameworks for spatial analysis of large tissue cohorts are still lacking. Both the size and the heterogeneity of the organ pose notable analytical challenges, including how to infer molecular information at the single-cell level, how to integrate imaging and molecular data and how to perform biologically meaningful comparisons between functionally relevant tissue domains across space and time. For example, many spatial analysis methods reduce noise by smoothing gene expression data across neighboring spots or cellular neighborhoods at the risk of true signal loss^34–36^. Most methods for testing spatial differential expression (DE) through clustering^37,38^ or explicit modeling^39,40^ are applied only to single tissue sections and those that integrate data from multiple tissue sections^41–44^ are usually limited to the alignment of serial sections from the same tissue. Other methods, focused on deconvolution of multicellular spatially resolved measurements to the single-cell level (through Bayesian modeling^45–47^, non-negative matrix factorization (NMF)^48^ or deep learning^49^), typically use little or no information about tissue anatomy or histology, which may yield biologically unrealistic results and limit their efficacy^50,51^. Furthermore, while a common coordinate system^52^ can facilitate integration, it is more challenging in large tissues such as the colon that lack a strict stereotypical architecture. Thus, to characterize the molecular and cellular variation underlying functional variation in the colon, both spatial profiling of large tissue cohorts and computational means to integrate these data across space and age are needed.

Here, we created a comprehensive experimental and computational framework to construct an integrated cell and tissue atlas of the mouse colon across temporal, anatomical and morphological variation, by combining spatial transcriptomics (ST)^31,53^ and single-nucleus RNA sequencing (snRNA-seq)^17,54^. We define the relative abundance of cell types using multimodal estimation, address missing data imputation and technical noise correction through information sharing across tissue sections and use explicit, hierarchical modeling of spatial and covariate-specific effects on cellular gene expression for Bayesian hypothesis testing across cell types, tissue regions, ages or other covariate groups informed by both snRNA-seq and ST data. Our work provides insights into tissue-level and cell-level function and organization and serves as an important reference for understanding the biology of aging.

## Results

### A spatiotemporal atlas of the colon

**Fig. 1:**
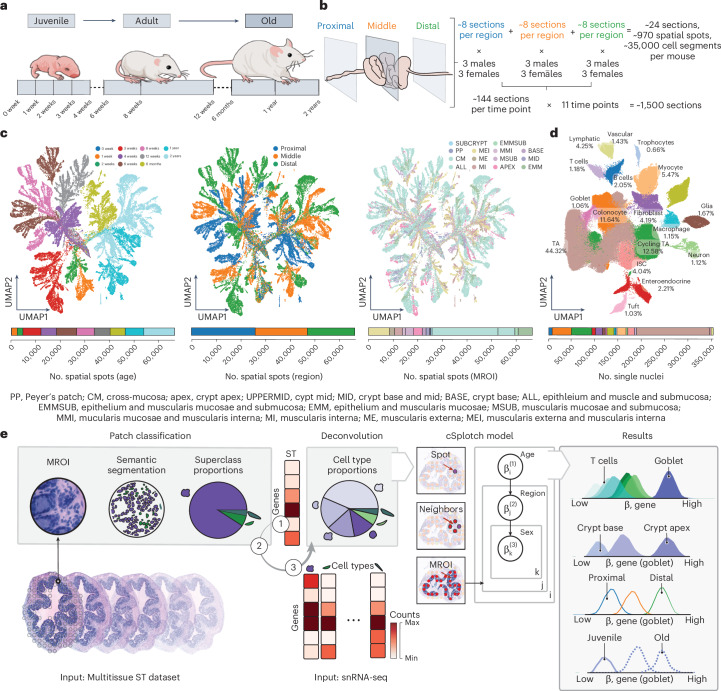
A cellular and spatial atlas of the mouse colon across regions and ages. Study design overview. **a**, Sampling time points: birth/juvenile (0–4 weeks), adulthood (6–12 weeks) and aging (6 months–2 years). **b**, Sampling regions: proximal, middle and distal colon. **c**, Spatial atlas. The *t*-distributed stochastic neighbor embedding (t-SNE) embedding of 66,481 ST spot profiles colored by age group (left), colon section (center) and MROI (right), along with relative abundances (stacked bars; bottom). **d**, Single-nucleus atlas. The t-SNE of 352,195 snRNA-seq profiles colored by cell type cluster (top) and their relative abundances (stacked bars; bottom). **e**, Analysis. Multitissue dataset (bottom left) served as input to cSplotch, which uses histological (MROI and semantic segmentation) and expression (ST and snRNA-seq priors) data to estimate the abundance of each snRNA-seq cell type in each spatial spot and then uses hierarchical Bayesian modeling (cSplotch model) to infer cell-type-specific gene expression conditioned on age, region, sex and MROI annotation (results).

**Extended Data Fig. 1:**
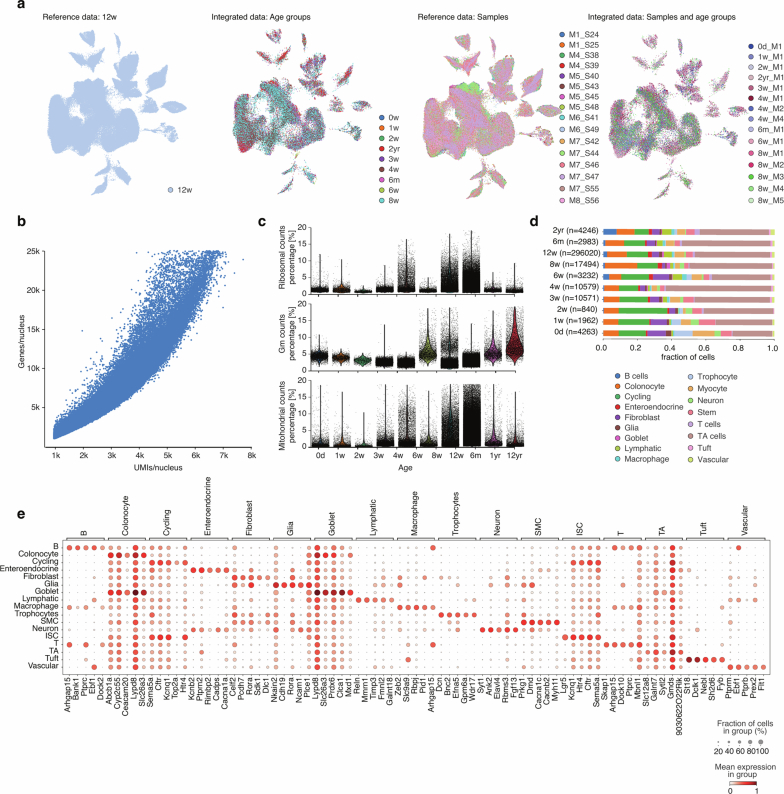
snRNA-seq data quality metrics and cell type associated genes. (**a**) UMAP representations of ~400,000 single nuclei. Newly generated data was integrated (**Methods**) into the reference dataset^17^ with all age groups and animal replicates color coded (left to right). (**b**) UMI vs. gene counts for ~400,000 snRNA-seq profiles used in this study. (**c**) Quality control metrics as percentages of ribosomal, Gm and mitochondrial gene counts detected in each age group. (**d**) Percentage of cell types (color code) detected in each age group. (**e**) Mean expression (dot size) and fraction of expression cells (dot color) of the top three marker genes (columns) for each of the 17 cell subsets (columns) defined from snRNA-seq.

To build a comprehensive atlas, we collected colonic specimens from proximal to distal anatomical regions through three major phases of the mammalian lifespan: juvenile (<4 weeks of age in the mouse), adulthood (6–12 weeks) and aging (6 months–2 years) and profiled them by ST^53^ and snRNA-seq^54^ (Fig. 1a). The spatial branch comprised ~1,500 sections (*n* = 65) and ~66,500 spatially barcoded spots, each quantifying the expression of 12,976 genes and sampling, on average, 24 tissue sections, 977 spots and 35,730 annotated cell segments from each mouse colon (Fig. 1b). These spanned 66 conditions across combinations of age (11 time points), sex (male and female), colonic region (proximal, middle and distal) and morphological regions of interest (MROIs; 14 histological annotation categories) (Fig. 1c and Supplementary Table 1). The cellular branch of the atlas encompassed a total of ~400,000 snRNA-seq profiles (*n* = 21), integrating newly generated and publicly available data, all collected in our lab (Extended Data Fig. 1a–c and Methods), which we partitioned and annotated into 17 major subsets of epithelial cells (intestinal stem cells (ISCs), transamplifying cells (TAs), cycling TAs, colonocytes, goblet cells, neuroendocrine cells and tuft cells), immune cells (B cells, T cells and macrophages), stromal cells (vascular cells, lymphatic cells, fibroblasts and trophocytes), enteric neurons, glia and smooth muscle cells (SMCs)^17,21,55^ (Fig. 1d, Extended Data Fig. 1d,e, Supplementary Table 2 and Methods).

### cSplotch infers cell type compositions and gene expression rates from ST, histology and snRNA-seq

To accurately detect spatial gene expression changes across multiple tissue samples, individuals and conditions in our atlas, we developed cSplotch^56^, a novel hierarchical Bayesian probabilistic model that uses both histological images and snRNA-seq to infer location-dependent and covariate-dependent cell-type-specific gene expression profiles from multicellular ST data (Fig. 1e). cSplotch consists of two major steps. First, it infers the cellular composition in each spatial spot from a multitissue dataset using a two-tier histological annotation schema, where MROIs, such as crypt apex and crypt base, are further labeled with cell-level morphological data, such as epithelial and muscle cells. These labels are then used for molecular integration with snRNA-seq profiles in a cell type deconvolution task. Second, cSplotch uses the deconvolved cell type compositions to infer MROI-specific and covariate-specific expression rates for each gene in each cell type. These expression rates can be used to test for DE across location (for example, crypt base versus apex), condition (for example, proximal versus distal colon or juvenile versus old mice), or cell type (for example, goblet versus T cell) in the entire atlas.

**Fig. 2:**
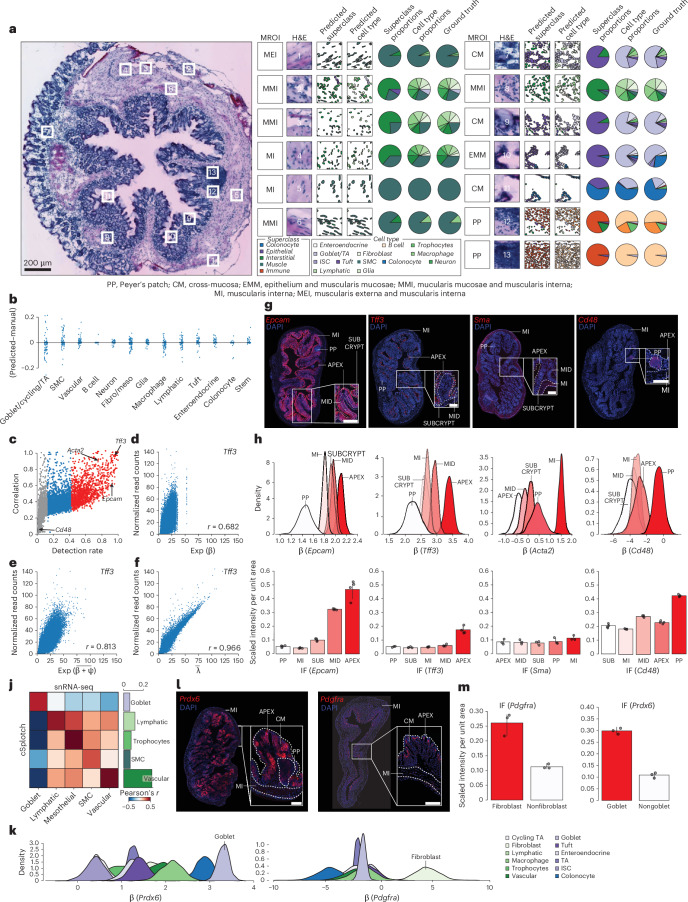
Deconvolutional modeling using histological and expression features. **a**,**b**, Agreement of morphology-informed deconvolution with manual cell type annotation. **a**, A total of 13 H&E patches from an example (8 weeks, distal) ST array (left; numbered boxes; scale bar, 200 μm) with their (from left) MROI annotation, H&E image patch, semantic segmentation of morphological superclasses (color code, bottom left) and snRNA-seq cell types (color code, bottom right) and relative proportions of predicted superclasses (pie charts), predicted cell types and manually labeled cell types (‘ground truth’). **b**, Error between predicted and manually calculated cell type fraction (*y* axis) for 13 cell types (*x* axis) evaluated across *n* = 88 manually annotated spots selected from 31 tissues (8 weeks) spanning all three colonic regions (11 proximal, 12 middle and 8 distal). **c**–**f** cSplotch model validation. **c**, Pearson correlation coefficients (*y* axis) between the expression rate estimated by cSplotch (λ) and TPM-normalized values in ST for the same spots scattered against the detection rate in ST (percentage of spots with nonzero measurements, *x* axis) for each of 12,796 genes (dots). Red and gray colors denote the top 10% and bottom 50% in detection rate, respectively. **d**–**f**, Variance in *Tff3* gene expression (*y* axis, normalized read counts) across spots (*n* = 66,481 dots) explained by region alone (**d**; mean ($\exp(\bar{\beta})$), *x* axis), region and location (**e**; mean ($\exp (\overline{\beta +\psi })$), *x* axis) or all components of the cSplotch model (**f**; mean $\bar{\lambda}$, *x* axis). Bottom right, Pearson’s *r*. **g**–**i**, IF validation of spatial gene expression trends predicted by cSplotch. **g**, IF of four gene products (*Epcam*, *Tff3*, *Sma* and *Cd48*, also highlighted in **c**) in proximal colon sections. Insets, zoomed-in views of a single colon crypt. Dotted lines denote the boundaries between MROIs: crypt apex, crypt mid, subcrypt, PP and MI (*Epcam*, *Tff3* and *Sma* scale bars, 200 μm; *Cd48* scale bar, 100 μm). **h**, Probability density (y axis) for posteriors over regional rate terms (*β*; *x* axis) inferred by the cSplotch model for the genes in **g** in each MROI (color code). **i**, Scaled IF intensities per unit area for each gene presented in **g** evaluated in at least three tissue sections (*Epcam*, Tff3 and *Cd48*, *n* = 4; *Sma*, *n* = 3). The color code is shared with **h**. Error bars, 95% confidence interval (CI); center, mean. **j**–**l**, Validation of the cellular expression profiles predicted by cSplotch. **j**, Cross-correlation between mean cell type expression profiles derived from two technologies: snRNA-seq (columns) and ST (cSplotch) (rows). The Pearson correlation (color scale) between mean external snRNA-seq profiles (columns) and mean cSplotch *β*s (rows) was calculated over the union of the top 50 cell type marker genes (Methods) for each of the top five cell types (by mean cell fraction) in the subcrypt for adult mice. **k**, Posteriors over cellular rate terms (β) inferred by cSplotch in CM in each abundant cell type (color code) for a goblet cell (*Prdx6*, top) and fibroblast (*Pdgfra*, bottom) marker in the CM region. **l**, IF images of proximal colon sections for the genes (*Prdx6* and *Pdgfra*) in **k**. Insets are the same as in **g**. Scale bar, 200 μm. **m**, Scaled IF intensities per unit area for each cell type presented in **l** evaluated in *n* = 3 tissue sections. Error bars, 95% CI; center, mean.

**Extended Data Fig. 2:**
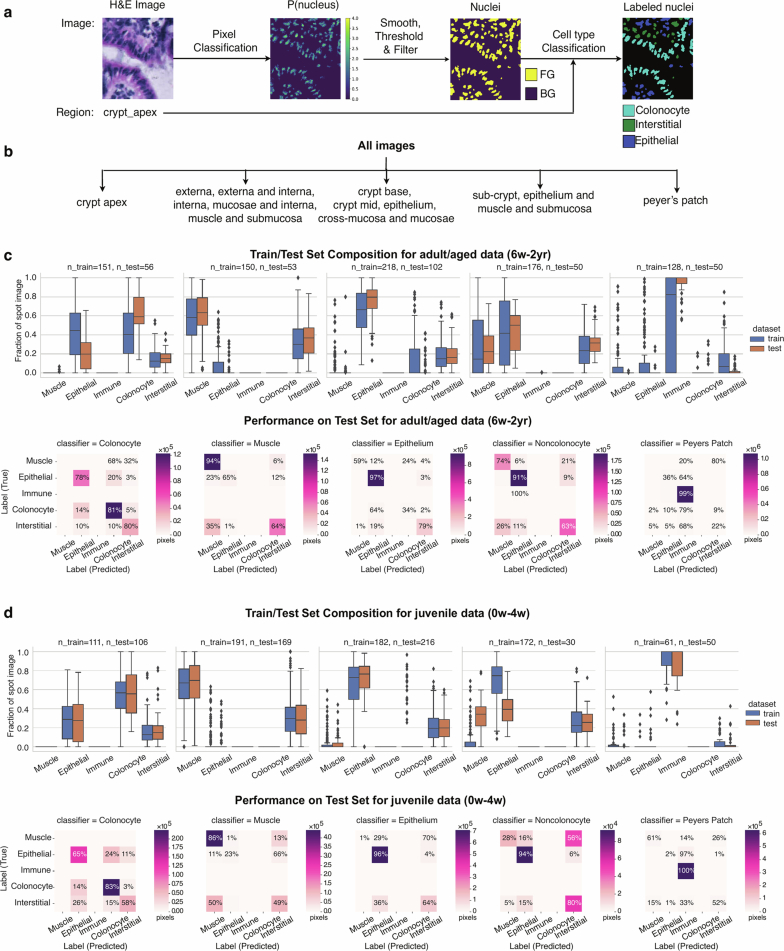
Validation of semantic segmentation of morphological cell types (“super-classes”). (**a,b**) Semantic segmentation workflow. Semantic segmentation of cell nuclei (**c**), conditioned on five broad annotation groups for spots (**b**). (**c,d**) Accuracy of cell type composition by semantic segmentation. Fractions of FG pixels in each H&E image (y axis) assigned to each morphological superclass (x axis) in test and train sets (color code) in adult (**c**, top) and young (**d**, top) mice, and percent and number (color scale) of pixels with a ground truth superclass label (rows) classified to each label (columns) in in adult (**c**, bottom) and young (**d**, bottom) mice. In Box plots: center black line, median; color-coded box, interquartile range; error bars, 1.5x interquartile range; black dots; outliers. Numbers of images used in train and test sets are denoted by “n_train” and “n_test”, respectively, above each subplot.

**Extended Data Fig. 3:**
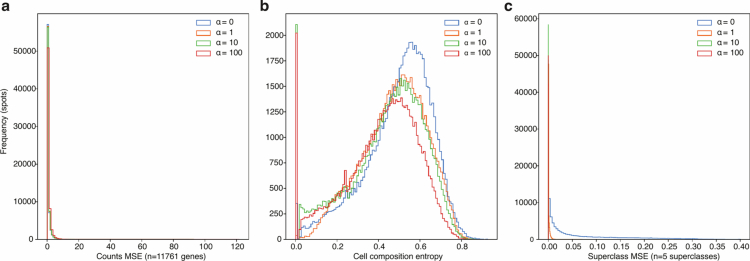
Effect of morphological constraints on count-based deconvolution of ST data with NMF. (**a**) Reconstruction of expression by NMF^48^ is unaffected by constrained weight parameter (α). Distribution (y axis) of mean squared error (MSE, x axis) between observed (N = 11761 genes) and SPOTlight-reconstructed expression (**Methods**) for N = 69,721 spots at different values of α (color code). **(b)** Cell compositions proposed by NMF decrease in entropy with increased α. Distribution (y axis) of entropy of cell composition vectors predicted by constrained NMF as in **(a)**. Entropy (x axis) is calculated across all 17 snRNA-seq cell types $({k}_{{sn}}=17$), and thus ranges between 0 (one cell type present) and 1 (all cell types present in equal proportion). (**c**) Reconstruction of morphological cell compositions is greatly enhanced with increased α. Distribution of residual MSE between predicted and observed morphological cell type (“superclass”) composition vectors from SPOTlight as in (**a**). Morphological cell type vectors are calculated from the output of SPOTlight by pooling snRNA-seq cell types from the same morphological class (${k}_{{morph}}$, as in Supplementary Table 3), reducing the space from ${{R}_{0\le x\le 1}}^{{k}_{{sn}}=17}$ to ${{R}_{0\le x\le 1}}^{{k}_{{morph}}=5}$.

Specifically, to infer cell composition, we first annotated each spatial spot with a single (mutually exclusive) MROI label (Methods), segmented nuclei from the histology image and annotated each nuclear segment with one of five morphological cell superclass labels (Fig. 2a), using a conditioned semantic segmentation workflow with structural, anatomical and neighborhood features (Extended Data Fig. 2, Supplementary Table 3 and Methods). Across held-out test sets for young (≤4 weeks old) and adult/aged (≥6 weeks old) mice and all anatomical regions, we correctly assigned 85% of all nuclear areas compared to pathologist superclass annotations as the ground truth (Extended Data Fig. 2c,d and Methods). Next, for each spot, cSplotch uses NMF^52,57^ to infer a combination of snRNA-seq profiles whose aggregate marker gene expression profile fits the observed expression measurement but in a manner constrained by the morphological cell composition inferred from histology in the previous step (Methods and Supplementary Table 4). Without introducing any morphological cell composition constraints (α=0), while gene expression profiles were well reconstructed (Extended Data Fig. 3a, blue line), the morphological cell type proportions were not, resulting in large numbers of deconvolved transcriptional cell types assigned to each spot (Extended Data Fig. 3b,c, blue lines). Once constrained by the inferred morphological cell type proportions (α > 0; Extended Data Fig. 3c, orange, green and red lines), the cell composition reconstruction improved, without loss of expression reconstruction accuracy. cSplotch performed well in both decomposing the expression profiles and recovering cell compositions consistent with pathologist annotations and known features of the tissue. For example, cell composition estimates for 88 spots randomly selected from 31 tissues (8 weeks) spanning all colonic regions (11 proximal, 12 middle and 8 distal) and MROIs agreed well with ground-truth pathologist annotations and well-known patterns of tissue organization in terms of cell type proportions (Fig. 2a,b). These included high SMC content in the muscularis regions, high goblet cell content in epithelial layers and high B cell content in Peyer’s patch (PP) (Fig. 2a).

**Extended Data Fig. 4:**
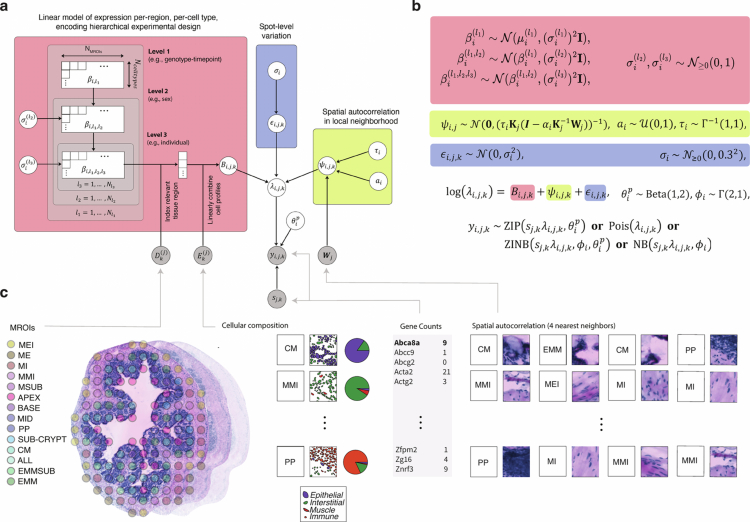
cSplotch statistical model for ST data. **(a,b)** Underlying statistical model of spatial expression for a gene *i* at spot *k* on tissue *j*. (**a**) Characteristic expression rate β (red shaded area) for gene *i* is inferred separately in each cell type and MROI (white rectangles). Stacked gray boxes: three-level hierarchical formulation of β (${l}_{1},{l}_{2},{l}_{3}$), where each level inherits from the one before it. Random variables ψ (yellow shaded area) and ε (blue shaded area) account for spatial autocorrelation and spot-level variation components, respectively. Gray and white circles: observed and latent variables, respectively. (**b**) Distributions over random variables of the statistical model in (**a**). ZIP, NB, ZINB: zero-inflated Poisson, binomial, and zero-inflated negative binomial distributions, respectively. (**c)** Model inputs. A multi-tissue colon dataset (left) is annotated at the spot level with MROI tags (left color key) and cellular compositions (segmentation masks, pie charts; lower color key), encoded as observed variables ${{D}_{k}}^{(j)}$ and ${{E}_{k}}^{(j)}$, respectively. The expression measurement at spot *k* (“Gene Counts”) yields the total sequencing depth of spot *k* (${s}_{j,k}$), and the observed counts (${y}_{i,j,k}$) of gene *i* (*for example*, Abca8a) therein. The (4-)neighborhood of each spot on tissue *j* (right) is encoded in the adjacency matrix ${W}_{j}$.

To infer gene expression rates, cSplotch next uses these inferred cell compositions, MROI annotations and sample covariates to fit a generalized linear model (GLM) of spatial gene expression across the atlas (Extended Data Fig. 4 and Methods). The model performs Bayesian inference to apportion the aggregate expression of each gene in each spot to the contributing cell type(s) in the spot (characteristic expression rate (β); hierarchically formulated to account for covariate-driven variation), along with the effects of neighboring spots (through spatial autocorrelation (ψ)) and spot-specific random effects (ϵ). The three hierarchical levels of the model—age, colon region and sex—are ordered by decreasing levels of hypothesized importance for gene expression variation. Additionally, to integrate the spatial samples in the context of a common coordinate framework, we used the 14 MROI categories that were manually assigned by the histology at each spatial spot (Methods).

**Extended Data Fig. 5:**
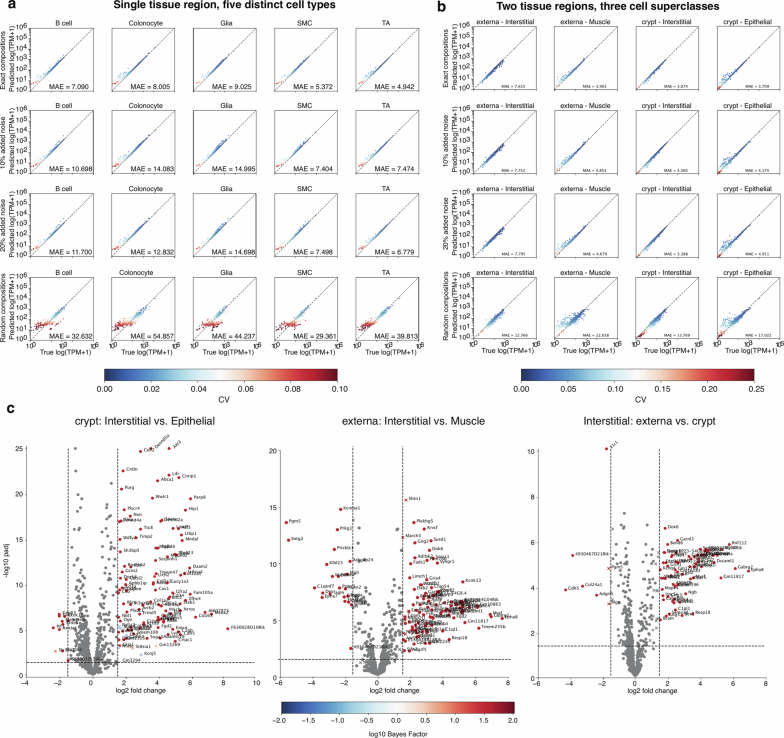
Validation of cSplotch deconvolutional capabilities on simulated ST data. (**a,b**) Correlations between true and predicted counts for simulated cell type clusters. Recovered (y axis, $1{0}^{6}\exp (\bar{\beta })$, where $\bar{\beta }$ is the posterior mean expression in a given cell type) and observed (x axis, TPM, snRNA-Seq data) expression for each of 1,000 genes (dots) with real input (top row) or at different levels of corruption of cellular composition input (other three rows) in cells from each of five superclasses (columns) from a single tissue region (**a**) or in each of two cell types from each of two different tissue regions (**b**). Bottom right: Mean absolute error (MAE). Dotted line: *x* = *y*. Color scale: coefficient of variation (CV; standard deviation / mean) for Splotch posterior estimates. (**c**) Differential expression effect size (log fold change; x axis) and significance (-log10 BH-adjusted p-value; one-sided t-test; y axis) for each gene (dot or X) between two cell types in one MROI (as labeled on top) based on snRNA-Seq data. Dots: genes showing agreement on DE assignment between snRNA-seq and Splotch X’s: genes showing disagreement on DE assignment. Dots/X’s for genes deemed DE from snRNA-seq (l2fc > 1.5, padj<0.05) colored by log10 Bayes factor (BF) of an analogous DE analysis between deconvolved cell profiles from cSplotch on simulated ST. Dashed vertical lines: LFC = |1 | ; dashed horizontal lines: p_adj_ = 0.05.

**Extended Data Fig. 6:**
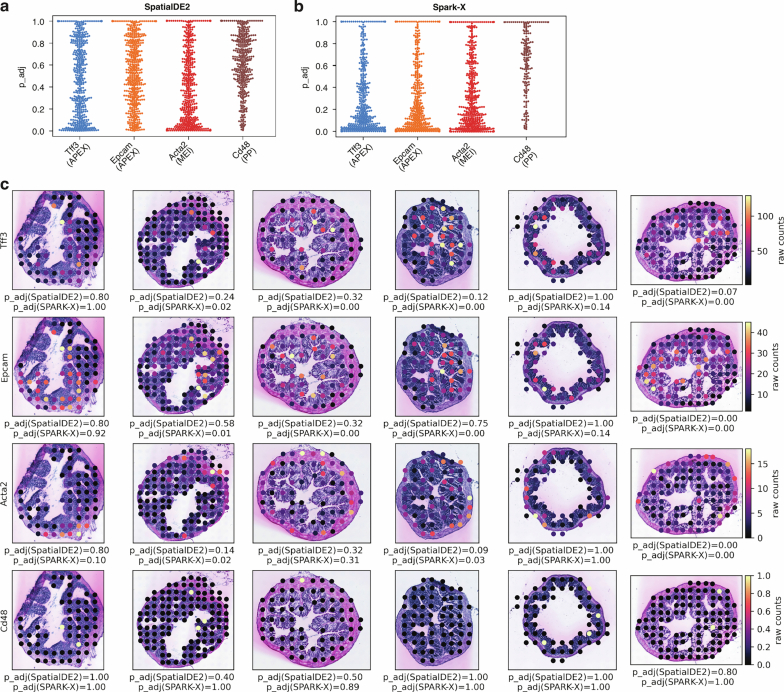
Comparison to SpatialDE2 and Spark-X. (**a-b**) Swarm plots showing spatial variability scores (p_adj) output by (**a**) SpatialDE2 (two-sided LR test (asymptotically Chi-square w/one degree of freedom); Benjamini-Hochberg FDR correction) and (**b**) SPARK-X (one-sided weighted sum of Chi-squares; Benjamini-Yekutieli FDR correction) methods for four genes (x-axis) across N = 734 tissue sections. Each gene is known to be localized to a specific MROI (parentheses) from IF imaging studies (Fig. 2g-i), and as such significant spatial DE is expected. (**c**) Observed spatial expression (color) of the same four genes (rows) on six tissue sections (columns) chosen at random from the dataset. For each gene on each tissue, adjusted *p-*values from SpatialDE2 and SPARK-X are denoted below the plot as p_adj (SpatialDE2) and p_adj (SPARK-X), respectively (identical test statistics and FDR correction to (**a**) and (**b**)).

**Extended Data Fig. 7:**
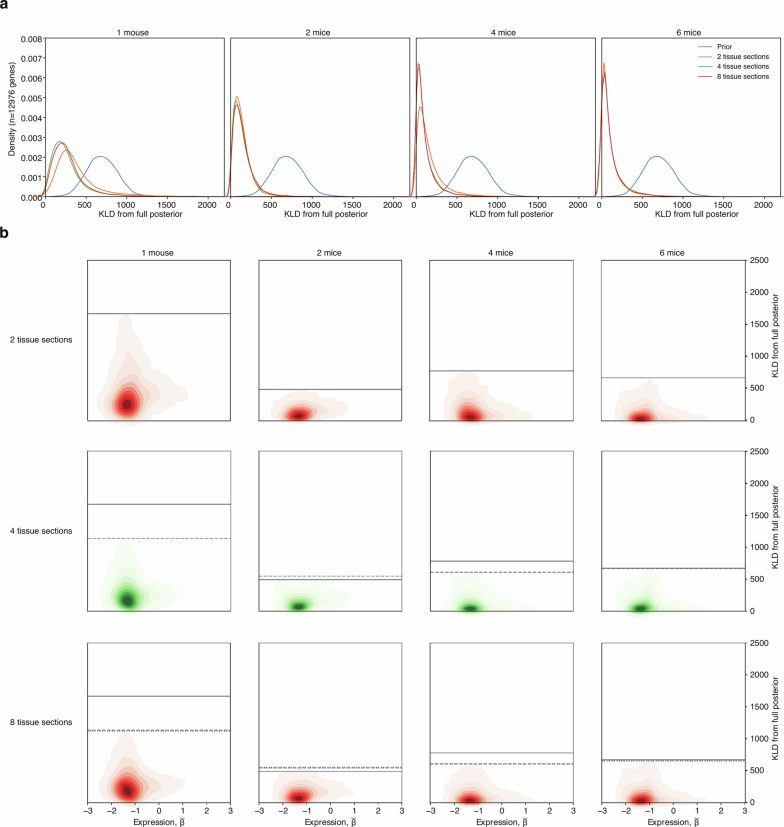
Effect of experimental design on study power. (**a**) Characteristic expression rates (β) in the cross-mucosa of the distal colon for 12-week-old mice. Distribution of Kullback-Leibler divergence (KLD, **Methods**) between posterior distributions of expression rates for n = 12,976 genes estimated from sub-sampled data (1, 2 and 4 mice; 2, 4 and 8 tissue sections per mouse) *vs*. the full data (6 mice, 53 tissue sections). Lower KLD values indicate greater agreement between full and subsampled data. **(b**) Mean expression $\bar{\beta }$ (posterior mean estimated from the full dataset) (x axis) and KL divergence (y axis) for each gene between the estimate from a sub-sampling of mice (columns) and tissue sections (rows) vs. the full data for each of n = 12,976 genes. For each number of subsampled mice, solid, dashed, and dotted lines represent maximum KLD value attained across all genes for increasing numbers of tissue sections.

**Extended Data Fig. 8:**
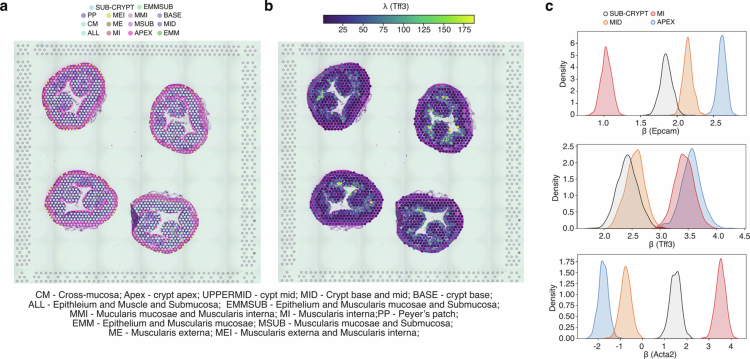
cSplotch applied to Visium from 10X Genomics. **(a)** Example Visium (10X Genomics) dataset of the proximal colon (age = 8 weeks). Spatial spots (color code) overlaid on top of the H&E image. **(b)** Inferred Tff3 gene expression (λ; color scale) overlaid on top of the H&E image as in **(a). (c)** Characteristic expression rates (β) for three spatial markers as in Fig. 2g-i for proximal 8-week-old mice.

We validated cSplotch’s inferred expression rates in our colon aging atlas data. Firstly, for highly expressed genes (~10% of the genes in the study, detected in >45% of all spots; Fig. 2c, red; for example, *Tff3*, *Ceacam1* and *Acta2*), the expression estimates of cSplotch were highly correlated to those obtained by transcripts per million (TPM) normalization and the spatial autocorrelation and spot-level variation components of cSplotch captured a substantial portion of the variance (Fig. 2d–f). Secondly, cSplotch recovered the correct spatial DE patterns in each MROI compared to immunofluorescence (IF) staining for four selected marker genes: the highly expressed *Epcam* and *Tff3* (enriched in crypt apex), *Acta2* (*Sma*; enriched in the muscularis interna (MI)), as well as *Cd48*, expressed in only 2.7% of spots (enriched in the PP) (Fig. 2g–i). Thirdly, the characteristic expression levels inferred for each cell type in a given MROI were most correlated with external snRNA-seq profiles from the expected cell type, for types present at both high (for example, vascular) and low (for example, goblet) frequencies (Fig. 2j). Fourthly, cSplotch correctly assigned genes with known cell-type-specific expression, such as *Prdx6* (goblet cells^58^) and *Pdgfra* (fibroblasts^59^), in the cross-mucosa (CM) and the muscularis externa (ME) and MI (MEI) region, confirming the accuracy of cSplotch in describing gene expression in a complex tissue region comprised of multiple cell types of varying proportions (Fig. 2k–m). Fifthly, cSplotch’s accurately deconvoluted simulated ST data, generated by constructing spots using mixtures of snRNA-Seq profiles (Supplementary Table 5 and Methods). cSplotch successfully reconstructed mean profiles for snRNA-seq (Extended Data Fig. 5a), morphological cell type clusters (Extended Data Fig. 5b and Methods) and DE patterns detected from snRNA-seq (Extended Data Fig. 5c and Methods; 94–100% agreement on DE genes), even when the cellular compositions used to deconvolve the simulated data were corrupted by Gaussian noise to reflect imperfect cell type annotations (Extended Data Fig. 5a,b). Sixthly, cSplotch performed better than SpatialDE2 and Spark-X, two methods^40,60^ that analyze ST data at the level of individual tissue sections, on the task of identifying spatially variable genes. Specifically, SpatialDE2 and Spark-X spatial DE statistics for four MROI-specific genes (*Tff3*, *Epcam*, *Acta2* and *Cd48*) vary greatly from section to section (Extended Data Fig. 6), with positive spatial DE determined only for a relatively low fraction of sections ranging from 1.3% (Cd48) to 15% (Tff3) in SpatialDE2 and 0.5% (Cd48) to 38% (Epcam) in Spark-X. These low detection rates may be because of the low signal-to-noise ratio and insufficient spatial sampling of individual tissue sections (particularly in PPs), limitations that cSplotch overcomes through hierarchical and multislice integration. Lastly, we estimated the impact of the number of individuals and tissues profiled on statistical power, by incrementally downsampling combinations of individuals (*n* = 1, …, 6 of 6) and tissue sections (*n* = 2, …, 8 of 52). Even when using one animal and two tissue sections, cSplotch captured meaningful expression signals, as reflected by the low Kullback–Leibler (KL) divergence from the posterior distributions derived using full data for that animal’s covariate group (age and region), and accuracy improved significantly with at least four mice (Extended Data Fig. 7a and Methods). Increasing the number of mice improved the estimation independent of expression level, whereas increasing the number of tissue sections per mouse improved the estimation of lowly expressed genes (Extended Data Fig. 7b and Methods). In our full dataset, we sample at least five mice per time point and a minimum of nine tissue sections per mouse, exceeding these thresholds. We confirmed the lesser importance of sex relative to age and colon region on expression variation, finding that at most 140 genes (0.01%) show DE across sex in any age or MROI (Supplementary Table 6) and that gene expression exhibits similar levels of variation within each sex group across all genes and conditions (coefficient of variation (5th, 95th) percentiles: (0.056, 5.46) for male and (0.057, 5.47) for female). Notably, cSplotch is compatible with higher-resolution ST technologies (for example, Visium and Visium HD; Methods) and recapitulates key spatial results (Extended Data Fig. 8 and Methods).

### Cell composition and cell-type-specific expression across the proximal–distal colonic axis correlates with functional variation

**Fig. 3:**
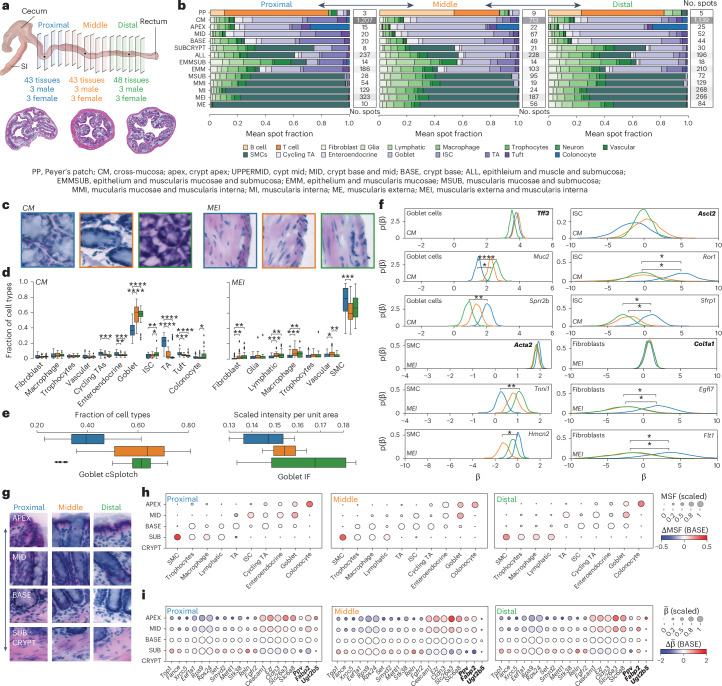
Regional differences in cell composition and function in the adult colon. **a**, The atlas spans structural variation in the mouse colon along the proximal–distal axis. Illustrative H&E images of sections from proximal (left; blue), middle (middle; orange) and distal (right; green) regions. **b**, Variation in cellular composition of MROIs across the proximal–distal axis. Proportion of cells of each type (*x* axis, stacked bars; color code) in each MROI (*y* axis) in the proximal (left), middle (middle) and distal (right) regions of adult (12 weeks) colon. The number of spots per MROI (color scale) in each colon region is indicated. **c**,**d**, Variation in cell type composition of the same MROI type along the proximal–distal axis. **c**, Representative H&E image patches from selected ST spots in two MROIs (left to right) from proximal (left; blue), middle (middle; orange) and distal (right; green) regions. In **d**, the fraction of cells (*y* axis) of each cell type (*x* axis) in the proximal (blue), middle (orange) and distal (green) regions is shown for each of the MROI types in **c**. Center black line, median; color-coded box, interquartile range; error bars, 1.5× the interquartile range; *0.01 < FDR ≤ 0.05, **10^−3^ < FDR ≤ 0.01, ***10^−4^ < FDR ≤ 10^−3^ and ****FDR ≤ 10^−4^ (Welch’s *t*-test). Only cell types observed at a rate of 2% or greater across all spots in an MROI are shown. **e**, Regional goblet cell abundances achieved with cSplotch as presented in **d** and those measured with IF (proximal and distal, *n* = 6; middle, *n* = 2). Center black line, median; color-coded box, interquartile range; error bars, 1.5× the interquartile range. The color code is shared with **d**. **f**, Cell-specific expression patterns vary across colon regions. Estimated posterior distributions of expression rate (β) for different genes in each colonic region (color) in goblet cells and ISCs in CM or SMCs and fibroblasts in MEI. Bold, canonical markers; brackets, significant DE (*BF > 2, **BF > 10, ***BF > 30 and ****BF > 100). **g**–**i**, Variation in structure, cell composition and gene expression along locations. **g**, Selected H&E image patches from ST across four MROIs (rows) from three colon regions (columns). **h**, Scaled (dot size) and absolute (dot color) change in mean spot fraction (MSF; dot color) of each cell type (columns) in each MROI (rows) relative to crypt base from three colon regions. **i**, Scaled (dot size) and absolute (dot color) change in mean log expression $(\bar{\beta })$ of each gene (columns) in each MROI (rows) relative to crypt base from three colon regions. Bolded genes, regional expression gradients.

To better describe the variation in tissue structure and function across the colonic tract, we first applied cSplotch to identify tissue-scale changes in cellular composition and spatial cellular gene expression along the proximal–distal axis in the adult (12 weeks) mouse colon. We analyzed 134 sections (43 proximal, 43 middle and 48 distal; 6,399 spots, ~55 nuclei per spot) across six age-matched and gender-balanced mice (three males and three females) (Fig. 3a). Relying on the classification in 14 MROIs, we estimated cell abundance and cell-type-specific expression of 17 distinct cell types.

A comparison of cell abundance in each MROI class across the proximal, middle and distal colon (Fig. 3b) showed widespread variation along the regions, especially in the transition from the proximal to the middle colon (Supplementary Table 7). Among the MROIs with the highest number of statistically significant differences in cell type abundance were the CM (significant regional differences in seven cell types) and the MEI (five cell types) (Benjamini–Hochberg-corrected Welch’s *t*-test, *P* < 0.05; Fig. 3c,d). In the CM, goblet cell proportions increased and TA frequencies decreased from the proximal to the distal colonic segment, whereas ISC proportions increased distally. In the MEI, SMC abundance declined distally, whereas fibroblasts, lymphatic cells, macrophages and vascular cells grew in frequency. These observations shed light on the cellular transitions associated with the change in colonic function along its longitudinal axis, with most digestive and absorptive functions occurring in the proximal colon, which handles final steps through peristalsis and bacterial enzymes, breaking down and absorbing residual starch and lipids, compared to water reabsorption, fecal compaction and storage in the distal colon. We compared our results to a previous estimate of cell type proportions along the human intestine, albeit by an earlier study that was not powered to statistically test longitudinal variation across subregions of the large tract^19^. Nevertheless, in both studies, goblet cells in the mucosa rose distally, whereas, in the MEI, SMCs increased and endothelial cells decreased in humans but not in mice. Thus, if adequately powered, a comparison between humans and mice may reveal both similarities and differences, which appear to follow known patterns of evolutionary variance at the anatomical and histological scale. In particular, while the mucosa cytoarchitecture is largely conserved, the external muscular layers are divergent across the two species^61^.

**Extended Data Fig. 9:**
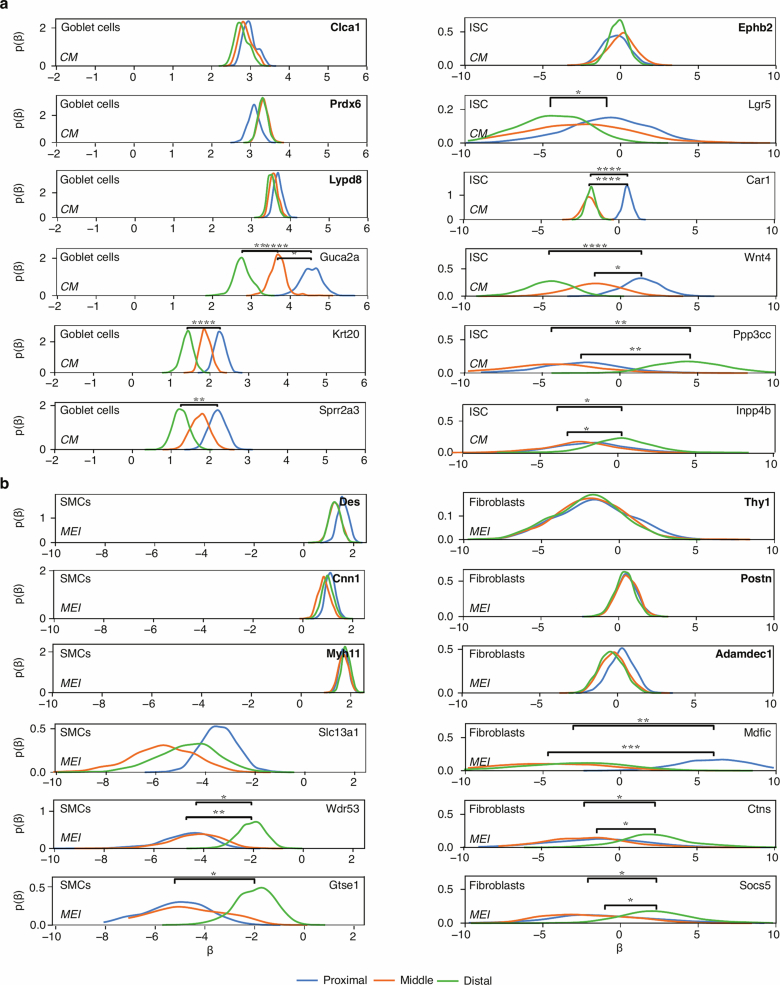
Effect of colon region on inferred cell-type specific expression distributions. Posterior distribution of characteristic expression rate (*β*; x-axis) of individual genes (labeled on top right) in each of the three regions (color) in goblet cells (left) and ISCs (right) in CM (**a**), and in SMCs (left) and fibroblasts (right) in MEI (**b**). **Bold**: Canonical markers. Brackets: significant differential expression (*: BF > 2; **: BF > 10; ***: BF > 30; ****: BF > 100).

**Extended Data Fig. 10:**
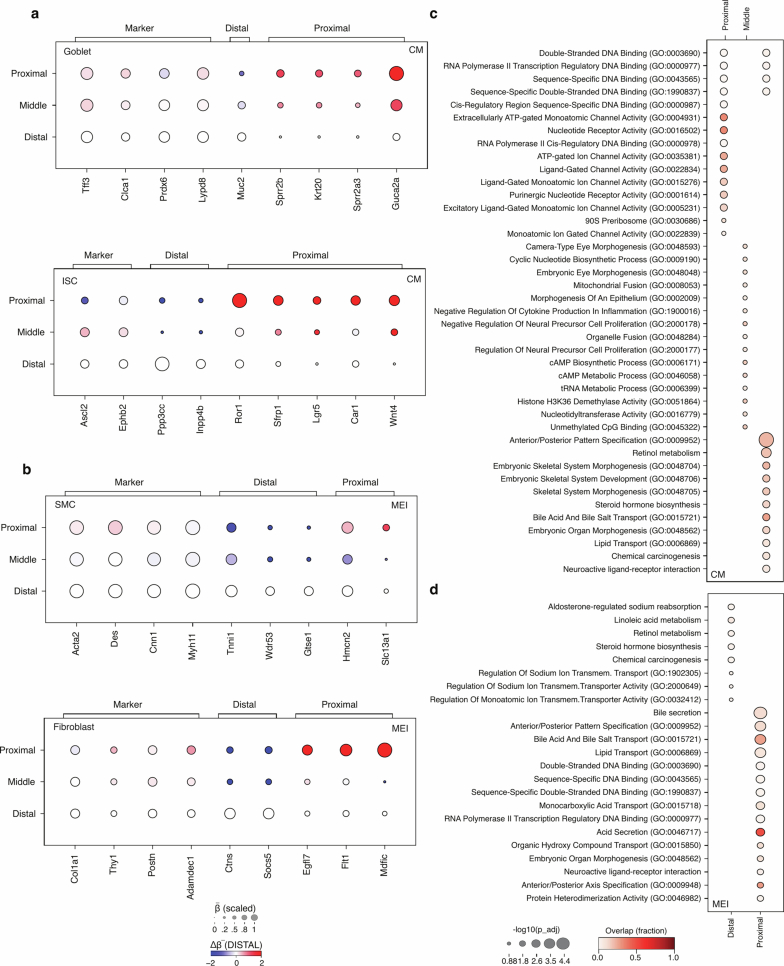
Effect of colon region on inferred cell-type specific expression and associated pathways. Scaled (dot size) and absolute (dot color) change in mean log expression ($\overline{\beta }$) of each gene (columns) in each colonic region (rows) relative to the DISTAL colon in goblet cells and ISCs in CM (**a**), and in SMCs and fibroblasts in MEI (**b**). Brackets denote markers and region-specific genes. (**c-d**) GSEA analysis for regional DE genes marked by cSplotch in (**c**) CM and (**d**) MEI. Dot size: -log10(p val). Color scale: Overlap between regional DE genes and GSEA process.

At the level of cellular gene expression, cSplotch analysis showed that canonical cell-type-specific markers and processes are similarly expressed across the three colonic regions, according to the posterior distributions of cellular expression rates (Fig. 3f, bold, Extended Data Figs. 9 and 10 and Methods), with stronger cell-type-specific signals than snRNA-seq alone (Supplementary Fig. 11) (*Tff3*, *Clca1* and *Lypd8* for goblet cells^62,63^; *Ascl2*, *Lgr5* and *Ephb2* for ISCs^64–66^; *Acta2*, *Cnn1* and *Myh11* for SMCs^67^; *Col1a1*, *Thy1* and *Postn* for fibroblasts^68^), although we acknowledge that this effect may be influenced by snRNA-seq dataset size and/or quality. Other genes had significant cell-type-specific regional differences (Bayes factor (BF) > 2) across the proximal–distal axis (Fig. 3f, nonbold, and Extended Data Fig. 9a,b). For example, in the CM, goblet cells upregulated the antimicrobial genes *Sprr2b* and *Sprr2a3* (ref. ^69^) proximally and the key gel-forming mucin *Muc2* distally. Such goblet-cell-specific expression patterns are consistent with the role of the proximal colon in controlling microbial fermentation and the requirement for a thick mucus layer as a protective barrier distally^70^. Within the same MROI, ISCs expressed proximally higher levels of canonical Wnt inhibitors (for example, *Sfrp1*) and mediators of the noncanonical pathway (for example, *Ror1* and *Wnt4*). As the strength of canonical Wnt signaling is tightly regulated locally and associated with ISC self-renewal^71^, the proximal colon may restrain this pathway and experience a slower cellular turnover. Conversely, the distal colon may require less restrained canonical Wnt signaling to replenish an expanded goblet population, which has the shortest lifespan across mucosal cell types^72^. Such observations are in line with embryological studies on gastrointestinal patterning demonstrating a key instructive role for an anterior-to-posterior Wnt signaling gradient^73–75^. In MEI, SMCs upregulated *Hmcn2* proximally and *Tnn1* distally. As *Hmcn2* is associated with Hirschsprung’s disease^76^ and *Tnn1* helps enforce contractility^77^, their regional-specific expression can support autonomous peristalsis proximally and voluntary contraction distally. In MEI, genes encoding for repressors of vasculogenesis and lymphogenesis, such as *Egfl7*, *Flt1* and *Mdfic*^78,79^, were upregulated by the fibroblasts proximally, indicating the loss of such mediators as potential mechanism for the expansion of the vascular system distally, where it is required for water reabsorption. Consistent with this, gene set enrichment analysis (GSEA; Methods) of genes differentially expressed between the proximal and distal regions in the CM and MEI showed an enrichment for pathways associated with lipid and sugar metabolism proximally and for ionic exchange distally, while those associated with voluntary contractility, as opposed to autonomous peristalsis, were enriched at the terminal colon (Extended Data Fig. 10c,d). Comparison to the Human Protein Atlas^58^ revealed that 128 of the regionally regulated genes, such as *Tff3*, *Clca1*, *Lypd8*, *Muc2*, *Ascl2*, *Ephb2*, *Myh11* and *Cnn1*, are among the 949 protein markers overrepresented in the human colon from a cohort of healthy individuals, suggesting a conservation of regional expression and likely function across species.

Collectively, we found that the variation in both cell abundance and cell-type-specific gene expression along the colon longitudinal axis correlates with distinct digestive functions along the colonic tract.

### Cell type and gene expression gradients along the crypt axis associated with colonic regeneration and functional differentiation

The intestinal crypt is responsible for the continuous regeneration of the highly specialized colonic mucosa^3^. As ISCs divide at the bottom of the crypt, they migrate toward the apex, differentiating into the main specialized lineages (goblet cells and colonocytes), as well as multiple rarer cell types, including enteroendocrine and chemosensory cells. A morphogen gradient modulates the balance between epithelial proliferation and differentiation but its composition at different biological scales is not fully characterized^80^. To date, targeted imaging approaches have characterized only a limited number of positionally restricted signaling mediators regulating crypt structure and function^81^.

Both cell morphologies and identities displayed distinct zonation patterns across four MROIs of the crypt axis (subcrypt, crypt base, crypt mid and crypt apex) (Fig. 3g,h). The proportions of ISCs, TAs and cycling TAs decrease gradually from the crypt base to apex, goblet cell proportions follow an opposite gradient, colonocyte abundance peaks sharply in the apex and enteroendocrine cells are evenly scattered across the crypt axis. SMCs are almost entirely confined to the subcrypt, whereas lymphatic cells, trophocytes and macrophages gradually decline in a base-to-apex direction. These cell-type-specific distributions are consistent with distinct positional dependencies. Colonocytes and SMCs are localized in close proximity to physical barrier domains (for example, the colonic lumen and the lamina propria), whereas the distribution of epithelial cells (ISCs, TAs and goblet cells), immune cells (macrophages) and some mesenchymal cells (trophocytes and lymphatic cells) is consistent with a reliance on overlapping signaling cues from the opposite crypt poles. Lastly, the homogeneous scattering of enteroendocrine cells may reflect the distribution of enteric nerve fibers.

At the molecular level, 321 genes had significant, monotonic variation across the crypt regardless of the colonic region (Fig. 3i, Supplementary Table 8 and Methods). Some of these genes were significantly associated (BF > 2, log_2_ fold change > 0.5; Methods) with specific cell types in a given spatial niche. For example, many of the genes upregulated in BASE were both expressed in ISCs and implicated in key biosynthetic pathways, including DNA replication and repair (*Top1*, *Fance* and *Xrcc5*), protein synthesis (*Eef1a1*, *Rps9* and *Rps24*) and transcriptional (*Set* and *Smyd2*), translational (*Mettl1*) and posttranslational (*Stk38*) regulation (Supplementary Table 9). Elevated biosynthetic activity restricted to basally located progenitors has also been observed across the human intestine of healthy individuals^19^ and in CRC^82^. Genes upregulated at the crypt apex were associated with the establishment of an apical polarity domain at the plasma membrane of goblet cells, including cytoskeletal (*Ezr*), cell–cell adhesion (*Ceacam1* and *Cldn3*) and transmembrane transport (*Slc26a3* and *Slc6a8*) genes (Supplementary Table 9). Additionally, receptors (*Fgfr2*) and ligands (*Reln*) mediating the crosstalk between ISCs^83–85^ and the underlying stromal compartment (for example, lymphatic cells and trophocytes) were upregulated in the subcrypt and crypt base. Consistent with this, GSEA showed an enrichment of genes from pathways associated with ribosomes and protein synthesis in the subcrypt, whereas genes associated with tight junctions, nutrient absorption and energy metabolism genes were enriched in the crypt apex (Supplementary Fig. 12a, Supplementary Table 9 and Methods).

Another 118 genes had crypt-oriented gradient expression preferentially in one colonic region (23 proximal, 53 middle and 42 distal). These included genes related to key metabolic functions, including the detoxifying enzyme *Ugt2b5*, which is downregulated in experimental models of colitis^86^, the fatty-acid-binding and importer protein *Fabp2* (ref. ^87^) and the gut hormone *Ppy*, known to regulate food intake^88^ (Fig. 3i). Consistent with this, genes differentially expressed specifically in the proximal, middle or distal crypt were enriched (by GSEA) for biosynthetic programs in the subcrypt. For IGF signaling, which is implicated in ISC biology^89^, DE genes were enriched in proximal crypts (Supplementary Fig. 12b–d). For programs related to vitamin C, DE genes were enriched in the proximal crypt apex. For vitamin A, DE genes were enriched in the middle colon crypt apex. Thus, each of the three colonic regions may rely on partially distinct molecular gradients associated with different digestive functions. Several such genes, especially those expressed in the highly specialized cells in the crypt apex, such as *Ceacam1*, *Cldn3*, *Slc26a3* and *Fabp2*, also display higher expression in the human colonic lumen^19,58^. A small subset of genes presented sex-biased expression across the crypt, including *Guca2a*, encoding the intestinal hormone guanylin^90^, and *Apoa1* and *Cyp3a44*, involved in cholesterol and steroid metabolism^91,92^, respectively (Supplementary Table 6).

In summary, our analysis revealed cell-type-specific distributions, likely reflecting distinct positional dependencies, and identified a subset of pan-colon or region-specific gradient genes to prioritize mechanistic experiments on the crypt’s structure and function.

### Spatiotemporal variability of cell type abundances during colon aging

In the elderly human population, the large intestine is affected by common pathological conditions including constipation^93^, diverticulitis^94^ and malnutrition^95^, a markedly increased risk of CRC^96^ and other features of intestinal senescence^8,18,97–103^. Yet, aging is a loosely defined condition, which is asynchronously experienced across populations with vastly variable phenotypic impact. Previous studies carried out on small cohorts with few time points, using destructive methodologies, have led to potentially conflicting findings. For example, both loss and gain of secretory cells have been reported to occur in the aging mouse intestine^18,102,104^.

To characterize intestinal cells during mouse aging, we tracked changes in the proportion of the most abundant cell types (ISCs, TAs, goblet cells and colonocytes) in the four crypt MROIs in each of the colonic regions over time (Supplementary Fig. 13 and Supplementary Table 10). During the juvenile (up to 4 weeks) and adult (4 to 12 weeks) phases, there was limited variation in the ISC fraction over time in the subcrypt, crypt base and crypt mid, consistent with the highly regenerative nature of the colon, both during development and at steady state. In the same compartments, TAs displayed a transient increase during the first 4 weeks after birth, followed by a decline between 6 and 12 weeks, as they increasingly transitioned toward the secretory lineage. Throughout juvenile and adult life, goblet cells steadily increased across all MROIs, reaching their peak proportion between 8 and 12 weeks. Although the structure and function of colonocytes is impacted by weaning from lactation to solid food starting 3 weeks after birth^105–107^, their relative proportion progressively decreased in the crypt apex up to 12 weeks, concomitantly with the increase in goblet cell fraction.

Importantly, there were multiple significant changes in cell composition of the same region between full reproductive maturity (12 weeks) and end of life (2 years) across the crypt axis in the distal and middle colon, as well as, to a lesser extent, in the proximal region (Supplementary Fig. 13, shaded areas, Supplementary Table 10 and Methods). Goblet cell frequency declined from young adult (12 weeks) to aged (2 years) animals in all MROIs of the crypt axis in the mid colon, in the subcrypt, crypt base and crypt mid regions in the distal colon and in the crypt base of the proximal colon. Conversely, the frequency of progenitor cells (ISCs and TAs) increased in old mice (2 years) in both the middle and the distal colon, with TAs increasing with age across the entire crypt axis in the middle colon and ISCs increasing with age in the subcrypt, crypt base and crypt mid MROIs in the distal colon. In the crypt apex of these segments (middle and distal), there was also a marked increase in colonocytes during that period. Hence, cell frequencies are substantially remodeled as animals age, with colonocytes progressively displacing a dwindling goblet population in the crypt apex. This may be especially impactful in the middle and distal mucosa, which rely more heavily on secretory cells at steady state. Goblet cells decline despite the concurrent increase in the proportion of ISCs and TAs in the subcrypt, crypt base and crypt mid. These temporal dynamics suggest an imbalance between progenitor proliferation and secretory differentiation, providing a cellular basis for the defective regenerative function observed in the elderly.

### Variation in cell type abundances associated with activity of multicellular programs (MCPs) during colon aging

**Fig. 4:**
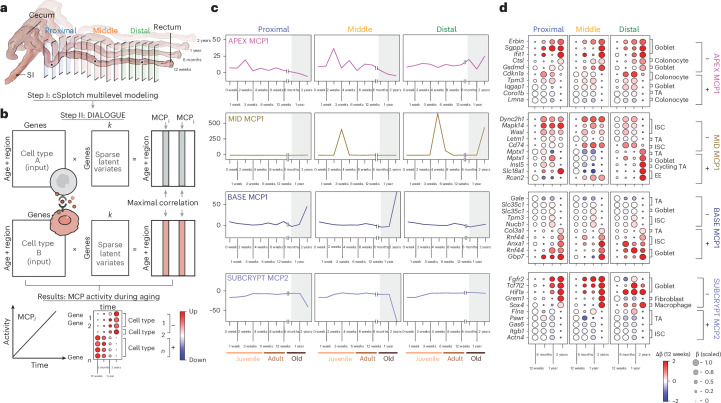
Spatiotemporal changes in cell composition and function during colon aging. **a**,**b**, Analysis approach. cSplotch was used to characterize variability in cell-type-specific gene expression across age and region (**a**), followed by MCP analysis with DIALOGUE^108^ (**b**) on inferred profiles for abundant cell types (for example, cell types A and B) in each MROI across ages and regions. MCPs are returned as sets of genes with high correlation between two or more cell types across conditions with positively (+, up) and negatively (−, down) contributing genes from each cell type (Methods). **c**,**d**, Aging-associated MCPs. **c**, Activity score (*y* axis) of selected MCPs from different MROIs (top left) for each time point (*x* axis) and region (top). Gray area: aging window. **d**, Scaled (dot size) and absolute (dot color) change in log expression $(\bar{\beta })$ relative to 12 weeks of each gene (rows) for each time point (columns) for selected upregulated (+) and downregulated (−) genes from the MCPs in **c**, sorted by their associated cell type (right).

To further identify expression changes potentially associated with distinct biological properties across covariate groups, we identified the genes with significant expression differences in the interval of 12 weeks to 2 years for every abundant cell type within each of the four crypt MROI (Supplementary Table 11) and tested the union of all DEGs (across all cell types) within each MROI for functional enrichments by GSEA. Enriched categories only included very broad terms, such as protein kinase signaling, regulation of transcription and vesicular transport, yielding limited insights into specific aging mechanisms (Supplementary Table 11). Because such gene-pooling approaches discard potentially useful information on the cellular source of expression and on cell–cell associations, we next characterized MCPs of genes with coordinated change in expression across multiple mucosal cell types with age. To that end, we applied DIALOGUE^108^, which uses multiple canonical correlation analysis (MCCA) to infer MCPs of genes with correlated expression patterns across multiple distinct cell types (Fig. 4a,b and Methods). Each MCP consists of subsets of genes whose mean expression in one cell type is positively or negatively correlated with that of genes in one or more other cell types across time (Supplementary Table 12). Several of the MCPs with the highest degree of correlation between member genes had near-monotonic changes during the aging window (for example, between 6 months and 2 years of age) in at least one colonic region (Fig. 4c, Supplementary Fig. 14, Supplementary Tables 12 and 13 and Methods).

Subcrypt MCP2, whose activity markedly declines at 1 year of age in the proximal and middle colon (Fig. 4d), may help explain some of the substantial changes in cell composition with age in the subcrypt region, where goblet cells decline and progenitors increase at 2 years of age (Supplementary Fig. 13). Specifically, subcrypt MCP2 consists of genes from nine cell types: epithelial (ISCs, TAs, cycling TAs and goblets), macrophages, trophocytes, lymphatic cells, vascular cells and SMCs. These include age-increasing proinflammatory genes in macrophages (*Sox4*)^109^ and signaling mediators in goblet cells (*Fgfr2*, *Tcf7l2* and *Hif1a*)^89,110,111^ and trophocytes (*Grem1*)^112^, many of which are involved in epithelial dedifferentiation^89,110,113^, inflammation^111^ and malignant transformation^112,114,115^, especially those associated with colitis^114,115^ (Supplementary Table 13). Age-declining genes included intestinal mechanotransduction (*Itgb1*, *Flna* and *Actn4*)^116–118^, cell death regulation (*Gas6* and *Pawr*)^119,120^ and tumor suppressor genes (TSGs; *Itgb1*, *Flna*, *Gas6* and *Pawr*)^116,119,121,122^ expressed in ISCs and TAs (Fig. 4d). As the adult distal subcrypt had enhanced Wnt signaling (Fig. 3f), the loss of expression of such TSGs could explain a shift in the balance between self-renewal and differentiation, leading to an expanded stem cell compartment and defective lineage priming. In contrast, the adult proximal subcrypt was enriched for canonical Wnt inhibitors. Here, TSG loss of expression alone may be insufficient to drive a wider progenitor compartment in the absence of a permissive microenvironment. Instead, we observed an upregulation of regenerative signaling mediators (*Fgfr2*, *Tcf7l2* and *Hif1a*), which may eventually promote dedifferentiation of the secretory lineage into progenitor cells. In line with such a hypothesis, the middle colon displayed an intermediate scenario, where the rewiring of goblet signaling was associated with an expansion of the TA fraction.

The activity of the crypt base MCP1 program, which consists of gene expression in ISCs, TAs and goblet cells, increases in the proximal and middle colon following 1 year of age. At 2 years of age, this MROI displayed loss of goblet cells throughout the colon and a gain in progenitors in the middle and distal colon. The crypt base MCP1 genes increasing with age included the key fetal stem cell marker *Anxa1* (ref. ^123^), upregulated in ISCs and associated with damage-induced regeneration^124^, *Rnf44* (ref. ^125^) and *Gbp7* (ref. ^126^), known to be induced by inflammation and expressed in ISCs and goblets, and the protumorigenic extracellular matrix protein *Col3a1* (refs. ^127,128^) found in TAs (Fig. 4d). Age-declining genes include metabolic regulators of glycosylated surface proteins and lipids, such as *Slc35a1* (ref. ^129^) and *Gale*^130^, which are important for immune recognition and pathogen infection, expressed in TAs and goblets, and *Tpm3*, a gene recurrently lost in CRC^131^, expressed in ISCs. The activation of damage-induced regeneration genes (*Anxa1*, *Rnf44* and *Gbp7*) suggests that injury-related responses are enhanced with aging predisposing the colon to transformation.

The crypt mid MCP1 program, with genes expressed in ISCs, TAs, goblet cells, enteroendocrine cells and tuft cells, had a different aging-related activity in the proximal and middle versus distal colon. At 2 years, goblet cells are lost and progenitors increase in the middle and distal crypt mid MROI, whereas no significant cell composition changes are observed in the proximal region. Crypt mid MCP1 genes with neuroendocrine and metabolic functions (*Insl5* (ref. ^132^), *Slc18a1* (refs. ^133,134^) and *Rcan2* (refs. ^135,136^)) declined with aging in the proximal and middle colon but increased in the distal colon. This suggested aging-related anteriorization of the longitudinal colonic patterning, an event also associated with the emergence of malignant states^137,138^. Such reprogramming genes were further coupled with the change in expression of genes involved in mucosal inflammation (*Cd74* and *Dync2h1*)^139,140^, stress response (*Mapk14* and *Letm1*)^141,142^ and CRC initiation (*Wasl*)^143^. The crypt mid MCP1 was also markedly enhanced in the middle and distal colon between 3 weeks and 4 weeks, at the time when juvenile mice undergo a drastic nutritional shift because of weaning, indicating that aging may aberrantly reactivate a stress state reminiscent of this important developmental transition.

Lastly, the activity of the crypt apex MCP1 program, which includes genes expressed in TAs, colonocytes and goblet cells, declined in the proximal and middle colon (Fig. 4c). MCP genes upregulated with age included inflammation genes related to pyroptosis (*Gsdmd*)^144^, proteolysis (*Ctsl*)^145^, membrane integrity (*Sgpp2*)^146^, cell polarity (*Erbin*)^147^ and the DNA damage response (*Ifit1*)^148^. Crypt apex MCP1 genes downregulated with aging included cytoskeleton regulators (*Coro1b*, *Iqgap1* and *Tpm3*)^131,149,150^ expressed in TAs, colonocytes and goblet cells, *Lmna*, a gene encoding the nuclear lamin A/C (whose loss is linked to genomic instability, whereas mutant isoforms are found in congenital premature aging syndromes^151–153^) and the cell-cycle repressor *Cdkn1a* (p21)^154^ (Fig. 4d). Thus, the aging colonic crypt apex is in a diffuse inflammatory state, associated with the expression of canonical aging markers and the downregulation of cell-cycle regulators, indicating the acquisition of a senescent state in the apical colonocytes coupled with the loss of tumor suppressor mechanisms.

## Discussion

Characterizing spatial patterning in large organs requires us to harmonize and relate molecular and morphological profiles measured by single-cell and spatial RNA-seq methods. Despite substantial progress to address spatial variance in expression and histological features, existing analysis methodologies are still mostly applied to a single tissue section at a time. As a result, they may fail to identify generalizable tissue function or identify how key features vary along anatomical, functional or temporal axes. Here, we developed a comprehensive experimental and computational framework and used it to present the first systematic atlas of colon aging.

Our analysis revealed changes in cell composition and cell-type-specific gene expression across multiple interleaving scales: gross anatomical scale (the longitudinal proximal–distal axis), fine histological scale (the crypt base to apex axis) and temporal scale (ages from newborn to old). Along the proximal–distal axis, these variations correlate with distinct digestive functions along the colonic tract. Along the crypt axis, we revealed cell-type-specific positional patterns and genes with either pan-colonic or region-specific spatially restricted expression across the main crypt axis. The positionally oriented balance between progenitor self-renewal and lineage differentiation was associated with a base to apex gradient between biosynthetic and cytoarchitectural regulators, prioritizing pathways and genes for functional validation. Along the temporal axis, aging was associated with a pervasive loss of goblet cells and the establishment of a diffuse inflammatory state, spanning expression programs in multiple cells and across the colon, and revealed the specific impact of such a scenario at different levels of the crypt axis and in distinct regions of the colonic tract. In the upper parts of the crypts, the progressive displacement of goblet cells by apical colonocytes or the anteriorization of the colonic metabolic patterning can provide molecular insights toward a mechanistic understanding of common geriatric conditions, such as constipation and malnutrition, paving the way for interventions aimed to support the quality of life and preventing systemic consequences. At the bottom of the crypt, increasing levels of damage coupled with persistent inflammation are associated with loss of TSG expression, excessive expansion of the progenitor compartment and cellular dedifferentiation toward fetal-like states. Such temporal dynamics are consistent with the elevated incidence of CRC premalignant states in the elderly and illuminate specific cell types and genes for guiding preventive and diagnostic strategies.

Using the canonical system of the mouse colon, we demonstrated how contextual spatial and temporal information can help decipher large-scale molecular datasets and how statistical models such as cSplotch can be used to connect tissue architecture with the pathological alterations leading to aging and disease. In this study, histology annotations were performed manually; however, this could be circumvented in the future with novel image classification approaches^155^. In the future, we plan to include additional modalities in our atlas, including protein expression to dissect immune cellular states and genome sequencing to measure mutational events and signatures, and increase temporal sampling frequency to further probe changing regenerative dynamics in elderly mice. We believe that this model framework has use in a wide range of tissue systems and hope that it may help to bridge the gap between single-cell and ST studies.

## Methods

### Image and ST data preprocessing

Hematoxylin and eosin (H&E) images were processed with SpoTteR (version 0.0.1)^156^. Briefly, original H&E images were scaled to approximately 500 × 500 pixels. Then, the tissue section was masked generously from the image through 10% quantile thresholding in a user-defined color channel. To detect probable spot centers, the image Hessian was computed. The spot centers then acted as potential grid points that were likely part of a regular grid structure and were selected by calculating the *x* and *y* distances between all detected centers. A regular grid was then fitted to these potential grid points using a custom optimizer based on the ‘nlminb’ function of the R package stats, which minimizes the distance of potential grid points to the suggested regular grid while assuming angles of 90° and 42 starting grid points per row and column. Through an iterative process, in which the 0.1% potential grid points that least fit the grid were removed in each iteration, the number of grid points per row and column was updated and a new grid was fitted until the target number of grid points per row (here, 35) and column (here, 33) was reached. Finally, those grid points that overlapped the tissue sections were identified by building a mask that represented the tissue area and registering all grid points that were present in this mask. In case a sectioning artifact was present, the corresponding ST spot was removed from all subsequent analyses.

### ST spot annotation

To assign each ST spot with a single (mutually exclusive) corresponding histological tag, a cloud-based interface^33^ was used to assign each spot (*x*, *y*) with one or more regional tags. A total of 14 tags (MROIs) based on established major gross morphology were used: crypt apex, subcrypt, crypt mid, crypt base, CM, epithelium and muscularis mucosae (EMM), EMM and submucosa (EMMSUB), epithelium and muscle and submucosa, ME, MEI, MI, muscularis mucosae and interna, muscle and submucosa, and PP.

### Detection of tissue sections in histology images

In most cases, more than one tissue section was placed on the active area of one ST array. To distinguish between different tissue sections, a two-dimensional integer lattice was assumed so that labeled ST spots that were connected were assigned the same tissue section. Next, ST spots were filtered on the basis of their sequencing data quality, such that tissue sections labeled with fewer than five (ages 0 days–3 weeks) or ten (ages 4 weeks–2 years) spots in total were discarded from further analysis. ST spots with fewer than 800 unique molecular identifiers (UMIs) were also discarded from further analysis. To account for spots without four neighbors, each spot was mapped after filtering to match the same two-dimensional integer lattice [[0,1,0], [1,1,1], [0,1,0]] and spots not matching this patterns were also discarded.

### Training a cell density classifier for segmentation

To train a cell density classifier to segment individual objects in the histology images, each whole-slide image (WSI) was first subset into smaller patches while retaining patches at the same resolution for training, selected such that each patch would contain all the major colon layers from at least one tissue cross section from the original WSI. Overall, ~200 patches were selected for training, with at least ten replicate patches from each of the different ages. To count the number of cell segments present in each ST spot, a density classifier was first trained using ilastik (version 1.4.1)^157^. This workflow estimated the density of blob-like structures usually present as overlapping instances, decreasing the chance of underestimating the number of objects because of undersegmentation, which we reasoned was the most appropriate approach for counting cell-dense areas in the colon. To ensure reproducibility across all density conditions in the dataset, in each training patch, at least three separate tissue areas (that is, training squares) were used. In each training square, two classes of objects were labeled: cells and background.

### Processing segments per ST spot

Each WSI processed with the SpoTteR ST processing tool^156^ was split into image patches of 200 × 220 pixels representing the size of an ST spot capture area. The cell-counting workflow described above was then used to extract density predictions for each ST spot. The following image processing and segmentation steps were performed with skimage^158^ (version 0.18.1). First, an ellipse shape (radius = 100 pixels) was used to mask the true ST capture area in each patch. If no cells were left in the patch (mean image intensity < 0.05), the patch was discarded from further processing. Next, the multi-Otsu^159^ thresholding algorithm (cutoff > 50) was used to separate objects detected in the patch. Local maxima were found for each object and used to estimate distances between the same. These were then approximated by the watershed algorithm^160^ into segments that were further labeled into individual objects used in all downstream analyses.

### Training an object classifier to obtain superclass cell type labels from histology images

MROI-specific object classifiers were trained using ilastik (version 1.4.1)^157^, with binary segmentation images and their corresponding H&E patches as input. In this way, each segment in the H&E patch was assigned a cell type superclass label. In each classifier, depending on the cells present in the corresponding MROI, up to five superclasses were labeled: colonocyte, immune, interstitial, muscle and epithelial. MROI-specific classifiers (colonocyte, PP, muscle, noncolonocyte and epithelium) with corresponding cell type superclass labels (colonocyte, immune, interstitial, muscle and epithelial), MROI labels and snRNA-seq cell type labels are presented in Supplementary Table 3. Because of differences in cell and tissue morphology, separate superclass classifiers were created for juvenile (0–4 weeks) and adult/aged (6 weeks–2 years) groups, for a total of ten image-based classifiers representing five MROI-specific classifiers across two age groups. To train each classifier, ~150 patches were randomly selected from all three regions of the colon (proximal, middle and distal) and from each of the following MROIs: CM, EMMSUB, subcrypt, crypt mid, crypt apex, MEI and PP. The object classifiers took into consideration object-level characteristics, such as object shape and work, to predict similar objects in the nearby space. Algorithm features used in training included two-dimensional convex hull and skeleton descriptors in a neighborhood size of 30 × 30 pixels for each object and used a simple threshold (0.5) with a small smoothing factor (1.0). Properties attributed to standard object features such as shape, size, channel intensity and location were also selected in the training process. In total, 1,540 patches and 83,721 segments were labeled during training.

### Processing cell type superclass from histology images per ST spot

Each H&E image patch (200 × 220 pixels) and corresponding segmentation predictions were used in ilastik batch processing to predict cell type superclasses using the object classifier described above. Cell label predictions were used in the following image processing workflow implemented using skimage (version 0.18.1)^158^. Each pixel class in the image was assigned one of the cell type superclasses. Then, small objects (<50 pixels) were removed from each patch and the remaining small segments abutting each other were merged if belonging to the same cell type class. The fractions of foreground pixels belonging to each object class were used as estimates of the abundance of each cell type in each patch.

### Testing the object classifier used for obtaining superclass cell type labels from histology images

To evaluate the performance of the cell superclass classifiers, a test set of 781 patches (50% size of training set) spanning the five adult/aged (colonocyte, immune, interstitial, muscle and epithelial) and five juvenile (colonocyte, immune, interstitial, muscle and epithelial) cell superclasses was set aside. Foreground objects were detected using the binary segmentation workflow, after which all objects were manually assigned to one of the five superclasses (colonocyte, immune, interstitial, muscle and epithelial). The same images were then input into the respective MROI-specific object classifiers and performance was evaluated by calculating the confusion (fraction of pixels labeled class A but predicted class B) for all pairs of cell types.

### Morphology-informed deconvolution using SPOTlight

The SPOTlight model was used for ‘bottom-up’ deconvolution of ST data^48^, which takes as input two matrices of count data: V, a (genes × cells) matrix containing the snRNA-seq count data (in which each cell is assigned to one of ${k}_{\mathrm{sn}}$ types), and $V{\prime}$, a (genes × spots) matrix containing the ST count data. Expression matrices were preprocessed in the following manner: (1) genes were subset to the set shared across both modalities; (2) data were depth-normalized to 10,000 UMI counts per cell or spot; and (3) data were scaled gene-wise to unit variance. Next, genes were further subset to cellular marker genes (log_2_(fold change) > 1, Benjamini–Hochberg false discovery rate (FDR) < 0.05; likelihood ratio test) and balanced across the ${k}_{\mathrm{sn}}$ cell types, selecting the top m=23 genes by FDR for each cell type, where m was chosen as the minimum number of significant marker genes across all cell types (23 for T cells). This resulted in a total of 334 unique genes used in deconvolution (Supplementary Table 4).

V was factored into component matrices W (genes × topics) and H (topics, cells) by NMF:

1 V=W×H

where the number of topics is assumed to be equal to the number of snRNA-seq cell types ${k}_{\mathrm{sn}}$. Before NMF, all values of ${W}_{g,t}$ were initialized to the probability of gene g being a marker gene for cell type t (quantified as Benjamini–Hochberg-corrected ${P}_{\mathrm{adj}}$ of *t*-test on log(count) data) and H was initialized as a binary matrix denoting the class assignment for each cell in the dataset. These initialization conditions—in which topics were treated equivalently to cell types—are meant to bias the optimization toward the discovery of biologically meaningful topic profiles.

Next, topic profiles W were fixed, and the following equation was minimized over $H{\prime}$ (topics, spots) by non-negative least squares (NNLS):

2 $$V{\prime} =W\times H{\prime}$$

In this manner, the expression profile of each spot in the ST data was mapped to a combination of topics inferred from snRNA-seq.

Third, Q, a (topics, ${k}_{\mathrm{sn}}$) matrix from H was derived by selecting all cells from the same cell type and computing the median of each topic for a consensus cell-type-specific topic signature. This topic matrix was used in a final NNLS minimization to find P, the (${k}_{\mathrm{sn}}$, spots) matrix denoting the inferred cellular composition of each ST spot:

3 $$H{\prime} =Q\times P$$

A modification to Eq. 3 was implemented that allows the incorporation of morphology-informed composition information derived from the image segmentation workflow, by providing two additional inputs: L, a (${k}_{\mathrm{morph}}$, spots) matrix containing the composition of each ST spot in terms of the ${k}_{\mathrm{morph}}$ morphological cell types defined in the segmentation model, and S, a (${k}_{\mathrm{morph}}$, ${k}_{\mathrm{sn}}$) binary matrix mapping each expression cell type to a morphological cell type. Any proposed compositional matrix P should additionally satisfy the following to reconstruct morphology-informed compositional data:

4 L=S×P

Morphology-aware SPOTlight decomposition was then achieved by solving the following optimization problem:

5 $$\mathop{\min }\limits_{P\ge 0}(H{\prime} -Q\times P)+\alpha (L-S\times P)$$

where α controls the relative importance of the morphological composition loss (second term) and the expression loss (first term). This optimization problem was solved using the PyTorch implementation of the Adam optimizer with a learning rate of 0.01 run for 100,000 iterations from a random initialization.

### cSplotch model specification

Genes *i*, tissue sections *j* and spots *k* were indexed as follows: $i\in [1,\cdots ,{N}_{\mathrm{genes}}],j\in [1,\cdots ,{N}_{\mathrm{tissues}}],k\in [1,\cdots ,{{N}^{(j)}}_{\mathrm{spots}}]$. Each tissue j was registered to a common coordinate system, such that each spot *k* was assigned to one of ${N}_{\mathrm{MROI}}$ distinct MROIs, denoted ${D}_{k}^{(j)}\in [1,\ldots ,{N}_{\mathrm{MROI}}]$, as described above. In the compositional mode, each spot was additionally assigned a simplex vector ${E}_{k}^{\,(j)}\in {{\mathbb{R}}}_{\ge 0}^{{N}_{\mathrm{cell}\,\mathrm{types}}}$, ${\sum }_{x}{E}_{k,x}^{\,(j)}=1\forall j,k$, which describes its proportional composition in terms of all ${N}_{{\mathrm{cell}}\,{\mathrm{types}}}$ unique cell types across the dataset.

For each gene and each spot, the observed counts ${y}_{i,\,j,k}$ were considered to be realizations of random variable with an expected value equal to ${s}_{j,k}{\lambda }_{i,\,j,k}$, where ${s}_{j,k}$ is a size factor (total number of UMIs observed at spot k) and ${\lambda }_{i,\,j,k}$ is the rate of expression of gene *i* (events per exposure), such that gene expression is modeled independently of sequencing depth. In practice, ${s}_{j,k}$ was further normalized by the median depth across all spots in the dataset to facilitate comparisons of results across analyses. Thus, cSplotch offers the user two choices for modeling count data: the Poisson distribution or the negative binomial (NB) distribution. Either may be supplemented with zero inflation to account for dropout events (technical zeros), yielding the zero-inflated Poisson (ZIP) or zero-inflated NB (ZINB) distributions:

6 $$\begin{array}{rcl}{y}_{i,j,k}\sim \{\begin{array}{ll}\mathrm{Pois}({s}_{j,k}{\lambda }_{i,j,k}) & \mathrm{nb}=0,\mathrm{zi}=0\\ \mathrm{ZIP}({s}_{j,k}{\lambda }_{i,j,k},{\theta }_{i}^{\,p}) & \mathrm{nb}=0,\mathrm{zi}=1\\ \mathrm{NB}({s}_{j,k}{\lambda }_{i,j,k},{\phi }_{i}) & \mathrm{nb}=1,\mathrm{zi}=0\\ \mathrm{ZINB}({s}_{j,k}{\lambda }_{i,j,k}{\phi }_{i},{\theta }_{i}^{\,p}) & \mathrm{nb}=1,\mathrm{zi}=1\end{array}\end{array}$$

where ${s}_{j,k}{\lambda }_{i,\,j,k}$ represents the expected mean of all distributions, ${\phi }_{i}$ represents the gene-specific overdispersion parameter for the NB family and ${\theta }_{i}^{\,p}$ represents the gene-specific probability of technical zeros or dropout. The zero-inflated model accounts for an overabundance of zeros by introducing a second zero-generating process gated by a Bernoulli random variable:

7 $$\begin{array}{l}{y}_{i,j,k}\sim \left\{\begin{array}{l}0\\ \mathrm{Pois}\left({s}_{j,k}{\lambda }_{i,\,j,k}\right)\end{array}\begin{array}{l}\mathrm{if}\;{\theta }_{i}=0\\ \mathrm{if}\;{\theta }_{i}=1\end{array}\right.\\ {\theta }_{i}\sim \mathrm{Bernoulli}\left({\theta }_{i}^{\,p}\right),{\theta }_{i}^{p}\sim \beta \left(1,2\right)\end{array}$$

where the Poisson process can be replaced by NB without loss of generality. This mixture model allows for ‘true’ biological zeros to be generated by the Poisson or NB process describing the expression model while ‘shunting’ technical zeros into a separate, technical process, preventing abundant dropout events from lowering the estimated mean expression ${\lambda }_{i,\,j,k}$. Because the Poisson process does not allow for overdispersion (variance exceeding the mean), ZIP should be preferred to Poisson in most situations, whereas the use of NB or ZINB may depend on data quality.

While cSplotch considered a separate random variable to describe gene expression in each spot, the rate parameters ${\lambda }_{i,\,j,k}$ were described in terms of a GLM that separates variation into shared and individual components. Specifically, the rate of gene expression was informed by three components:

8 $$\log ({\lambda }_{i,\,j,k})={B}_{i,\,j,k}+{\psi }_{i,\,j,k}+{\epsilon }_{i,\,j,k},$$

where ${B}_{i,\,j,k}$ describes the characteristic expression of gene *i* within the tissue context of spot k, ${\psi }_{i,\,j,k}$ describes the neighborhood effects and ${\epsilon }_{i,j,k}$ describes spot-specific effects. ${B}_{i,j,k}$ is calculated as a weighted sum of cellular expression rates ${\beta }_{i}$ in proportions ${{E}_{k}}^{(j)}$. Cellular expression was allowed to vary both across MROIs and sample conditions. As such, a characteristic expression matrix ${\beta }_{i}\in {{\mathbb{R}}}^{{N}_{\mathrm{MROI}}\times {N}_{{\rm{c}}{\rm{e}}{\rm{l}}{\rm{l}}{\rm{t}}{\rm{y}}{\rm{p}}{\rm{e}}{\rm{s}}}}$ was defined (when compositional data are unavailable, each MROI may be treated as composed of a single ‘average’ cell type and ${\beta }_{i}\in {{\mathbb{R}}}^{{N}_{\mathrm{MROI}}}$ is defined instead). Inferring a posterior over ${\beta }_{i}$ allowed the quantification of expression changes across regions or cell types by comparing relevant entries.

Because characteristic expression is expected to vary across conditions (for example, age, colon region and sex), region-specific expression ${\beta }_{i}$ was modeled in a hierarchical fashion defined by sample covariates. Up to three levels were explicitly modeled in the hierarchy, each of which split the sample to distinct groups along some covariate. At the top level, the dataset was split along an important covariate (for example, age) and a separate ${{\beta }_{i}}^{({l}_{1})}$ was modeled for each unique set ${l}_{1}\in \{1,\cdots {L}_{1}\}$. At the next level, each set was further partitioned along another covariate (for example, colon region). ${{\beta }_{i}}^{({l}_{1},{l}_{2})}$ was assumed to be centered around its corresponding top-level estimate ${{\beta }_{i}}^{({l}_{1})}$, with some additional variance associated with the new covariate ${\sigma }_{i}^{({l}_{2})}$. This encoded knowledge about the experimental system and separated out sources of variation associated with each covariate (without propagation of uncertainty, as all levels are inferred simultaneously). A three-level hierarchical model for ${\beta }_{i}$ was, thus, specified as:

9 $$\begin{array}{l}{\beta }_{i,{l}_{1}}{\mathscr{ \sim }}{\mathcal{N}}\left({\mu }_{i}^{\left({l}_{1}\right)},{\left({\sigma }_{i}^{\left({l}_{1}\right)}\right)}^{2}{\bf{{I}}}\right)\\ \begin{array}{l}{\beta }_{i,{l}_{1},{l}_{2}}{\mathscr{ \sim }}{\mathcal{N}}\left({\beta }_{{i,l}_{1}},{\left({\sigma }_{i}^{\left({l}_{2}\right)}\right)}^{2}{\bf{{I}}}\right)\\ {\beta }_{i,{l}_{1},{l}_{2},{l}_{3}}{\mathscr{ \sim }}{\mathcal{N}}\left({\beta }_{{i,l}_{1},{l}_{2}},{\left({\sigma }_{i}^{\left({l}_{3}\right)}\right)}^{2}{\bf{{I}}}\right)\\ {\sigma }_{i}^{\left({l}_{2}\right)},{\sigma }_{i}^{\left({l}_{3}\right)} \sim {{\mathcal{N}}}_{\ge 0}(0,1)\end{array}\end{array}$$

where, in the compositional mode, prior hyperparameters ${{\mu }_{i}}^{({l}_{1})}(\cdot ,m)$ and ${{\sigma }_{i}}^{({l}_{1})}(\cdot ,m)$ are set to the empirical mean and s.d., respectively, over the expression of gene *i* in cell type *m* in the snRNA-seq data for all MROIs; in the noncompositional mode (one ‘average’ cell type per MROI), ${{\mu }_{i}}^{({l}_{1})}(\cdot )$ and ${{\sigma }_{i}}^{({l}_{1})}(\cdot )$ are set to 0 and 2, respectively, for all MROIs. Variation parameters ${\sigma }_{i}^{\left({l}_{2}\right)},{\sigma }_{i}^{\left({l}_{3}\right)}$ are assumed to have truncated Gaussian priors reflecting our limited knowledge of the effects of covariate-driven variation and are inferred separately for each level 2 and 3 covariate group. For convenience, because each tissue j belongs to one covariate group at each level, the inverse mapping function ${\rho }^{-1}(j)$ was introduced to map j to the appropriate ${l}_{1},{l}_{2},{l}_{3}$ indices for ${\beta }_{i}$. ${B}_{i,\,j,k}$ was formally defined in the noncompositional model:

10 $${B}_{i,\,j,k}=\left\{\begin{array}{l}{x}_{j,k}^{T}{\beta }_{i,{\rho }^{-1}\left(j\right)}{E}_{k}^{\,\left(j\right)}\\ {x}_{j,k}^{T}{\beta }_{i,{\rho }^{-1}\left(j\right)}\end{array}\begin{array}{l}\text{if compositional}\\ \mathrm{else}\end{array}\right.$$

where ${x}_{j,k}^{T}$ is a one-hot encoding of ${{D}_{k}}^{(j)}$, the MROI annotation for spot *k*. With this framework for integrating multiple sections or experiments, the posterior distributions of the latent parameters ${\beta }_{i}$ were studied at different levels of the hierarchical experimental design and expression changes were quantified across conditions, tissue contexts or individual cell types.

The second component of Eq. 8, ${\psi }_{i,j,k}$, describes the effects of the local neighborhood of spot *k* on the expression of gene *i*. This was modeled using the conditional autoregressive (CAR) prior, which assumes that the value at a given location (spot) is conditional on the values of neighboring locations (spots). ${\psi }_{i,j}$ was defined as a Markov random field over the spots on each array:

11 $$\begin{array}{l}{\psi }_{i,k}|{a}_{i},{\tau }_{i},{{\bf{W}}}_{j}{\mathscr{ \sim }}{\mathcal{N}}\left({\bf{0}},{\left({\tau }_{i}{{\bf{K}}}_{j}\left({\bf{I}}-{{a}_{i}{{\bf{K}}}_{j}^{-1}{\bf{W}}}_{j}\right)\right)}^{-1}\right)\\ {a}_{i}{\mathscr{ \sim }}{\mathcal{U}}\left(0,1\right)\\ {\tau }_{i} \sim {\Gamma }^{-1}\left(1,1\right)\end{array}$$

where ${a}_{i}$ is a spatial autocorrelation parameter, ${\tau }_{i}$ is a conditional precision parameter, ${{\bf{K}}}_{j}$ is a diagonal matrix containing the number of neighbors for each spot in tissue j and ${{\bf{W}}}_{j}$ is the adjacency matrix (with zero diagonal). If the classic ST methodology of using cartesian arrays is applied, each spot is assumed to have a four-spot neighborhood; if the Visium platform using hexagonal arrays is applied, each spot is assumed to have a six-spot neighborhood. The level of spatial autocorrelation (${a}_{i}$) and conditional precision (${\tau }_{i}$) was inferred separately for each gene, although weak hyperpriors on both parameters are shared across all genes to enforce the same physiological constraints (Eq. 11). Taken together, the B and ψ terms capture spatial autocorrelation on two different scales: tissue context (across samples) and local neighborhood (within samples).

The final component of Eq. 8, ${\epsilon }_{i,\,j,k}$, captures variation at the level of individual spots. This variation was assumed to be independent and identically distributed for each gene:

12 $$\begin{array}{l}{\epsilon }_{i,\,j,k}{\mathscr{\sim }}{\mathcal{N}}\left(0,{\sigma }_{i}^{2}\right)\\ {\sigma }_{i}\sim {{\mathcal{N}}}_{\ge 0}\left({0,0.3}^{2}\right)\end{array}$$

where ${\sigma }_{i}$ is the inferred level of variability for gene *i*. The degree of variance in the truncated Gaussian prior on ${\sigma }_{i}$ is set to 0.3 following the work of Maniatis et al.^161^, who chose the value to reflect the relatively low levels of spot-specific variation (relative to other model terms).

### BF estimation for cSplotch DE analysis

To quantify difference in expression between two conditions using cSplotch, the BF between posterior distributions over characteristic expression coefficients β estimated by the model was examined. Without loss of generality, the difference in expression was quantified between conditions represented by ${\beta }^{(1)}$ and ${\beta }^{(2)}$, which may differ across any combination of genes, sample covariates (for example, distal versus proximal colon), tissue regions (for example, crypt apex versus muscle) or cell types (for example, neuron versus myocyte). A random variable ${\Delta }_{\beta }={\beta }^{(1)}-{\beta }^{(2)}$ was defined, which captures the difference between ${\beta }^{(1)}$ and ${\beta }^{(2)}$. If ${\Delta }_{\beta }$ is tightly centered around zero, then the two distributions are very similar to each other and the null hypothesis of identical expression cannot be rejected. To quantify this similarity, the posterior distribution ${\Delta }_{\beta }|{\mathcal{D}}$ (where ${\mathcal{D}}$ represents the data used to train the model) was compared to the prior distribution ${\Delta }_{\beta }$ using the Savage–Dickey density ratio that estimates the BF between the conditions:

13 $$\mathrm{BF}\approx \frac{p\left({\Delta }_{\beta }=0\right)}{p\left({\Delta }_{\beta }=0|{\mathcal{D}}\right)}$$

where the probability density functions were evaluated at zero. If expression is different between the two conditions, then the posterior ${\Delta }_{\beta }|{\mathcal{D}}$ will have very little mass at 0 and the estimated BF will be large (by convention, $\mathrm{BF} > 5$ indicates substantial support). Conversely, for similar expression regimes, the posterior will place a mass equal to or greater than that of the prior at zero and the BF will be ≤1. While $p({\Delta }_{\beta })$ can be derived analytically (the prior distributions over all β are normally distributed and the difference between two normally distributed random variables is in turn normally distributed), $p({\Delta }_{\beta }|{\mathcal{D}}{\mathscr{)}}$ must be approximated using the posterior samples obtained below. When we executed a comparison between sets of conditions (for example, neurons versus all other cells), we pooled the posterior samples from all component conditions together.

### Parameter inference for cSplotch

cSplotch was implemented in Stan^162^. For all analyses, Bayesian inference was performed over the parameters using Stan’s adaptive Hamiltonian Monte Carlo (HMC) sampler with default parameters. Four independent chains were sampled, each with 250 warmup iterations and 250 sampling iterations, and convergence was monitored using the $\hat{R}$ statistic. On average, sampling of a given gene was completed in ~3 h parallelized over four CPUs (<2 GB of total RAM used) and the sampling time for each chain was linear in both the number of spots in the dataset and the number of samples drawn, while multiple genes can be run in parallel. For high-resolution technologies (for example, Visium HD), the number of spots per tissue is dramatically increased and users may consider using variational inference to initialize HMC sampling (provided as ‘splotch-vinit’ executable) to reduce sampling time and/or suppressing the CAR model component (accounting for ~60% of computational time) with the ‘--no-car’ option.

### Simulated ST data generation

Simulated ST data were generated from the snRNA-seq profiles in the two regimes described in the subsequent sections. For all simulation studies, 12 ST arrays were generated, each containing 2,000 spots. For data in which distinct MROIs were simulated, the two regions were considered to exist in a 1:1 ratio. Cell clusters comprising each region are detailed in Supplementary Table 5.

#### Cluster-based simulation

Average expression profiles for unique mouse colon cell types obtained from snRNA-seq $G\in {{\mathbb{Z}}}_{\ge 0}^{{N}_{\mathrm{genes}}\times {N}_{{\rm{c}}{\rm{e}}{\rm{l}}{\rm{l}}{\rm{t}}{\rm{y}}{\rm{p}}{\rm{e}}{\rm{s}}}}$ (${N}_{\mathrm{genes}}=22,986,{N}_{\mathrm{cell}\,\mathrm{types}}=30$) were normalized column-wise, such that the total expression within each cluster summed to 1. For each spot k from tissue j, a true composition vector ${E}_{k}^{(j)}\in {{\mathbb{R}}}_{\ge 0}^{{N}_{\mathrm{celltypes}}}$ was drawn such that a random subset of cell types present in the current region (${D}_{k}^{(j)}$; Supplementary Table 5) were represented in uniformly random proportions. For each cell type t, reads ${y}_{k,t}$ were drawn from a multinomial distribution:

14 $${y}_{k,t}\sim \mathrm{Mult}\left({M}_{k}^{\,\left(j\right)}{E}_{k,t}^{\,\left(j\right)},{G}_{.,t}\right)$$

where ${M}_{k}^{\,\left(j\right)}$ is the total number of reads in the current spot and ${G}_{.,t}$ is the expression profile for cell type t. In practice, ${{M}_{k}}^{(j)}=1,000$ was used for all j,k. Read counts were then pooled across all cell types, yielding spot-level reads ${y}_{k}={\sum }_{t}{y}_{k,t}$. Composition vectors were then (optionally) pooled within high-level annotation categories (such as the histological superclasses in Supplementary Table 3) to simulate cases of limited observability. Finally, Gaussian noise was (optionally) added to ${E}_{k}^{\,(j)}$ to generate ‘observed’ composition vectors ${\widetilde{E}}_{k}^{\,(j)}$. Resulting negative entries were removed by element-wise maximization against the zero vector (${\widetilde{E}}_{k}^{\,(j)}\leftarrow \max ({\widetilde{E}}_{k}^{\,\left(j\right)},0)$), following which ${\widetilde{E}}_{k}^{\,(j)}$ was renormalized to produce a valid simplex. $y,D,\widetilde{E}$ served as inputs to cSplotch. Randomized compositions (highest level of noise) were generated by drawing from an ${N}_{\mathrm{cell}\,\mathrm{types}}$-dimensional uniform (0, 1) distribution and then unit-normalizing sums to produce valid simplexes.

For all cluster-based data, cSplotch was run with a Poisson likelihood (without zero inflation) to meet the expected form of the marginals over the generating distribution (multinomial). To focus on the compositional module of cSplotch, the effects of local spatial autocorrelation were removed by simulating each spot independently and, as such, suppressed the *ψ*_*i*_ term of the GLM. Local neighborhood effects can be simulated in this regimen by passing a spatial smoothing filter over the simulated array, blending the transcriptome of each spot with those of its neighbors in a defined proportion.

#### Cell-based simulation

Individual snRNA-seq cell profiles $G\in {{\mathbb{Z}}}_{\ge 0}^{{N}_{\mathrm{genes}}\times {N}_{{\rm{c}}{\rm{e}}{\rm{l}}{\rm{l}}{\rm{t}}{\rm{y}}{\rm{p}}{\rm{e}}{\rm{s}}}}$ (${N}_{\mathrm{genes}}=22,986,{N}_{\mathrm{cells}}=419,334$), where each cell was assigned to one of ${N}_{\mathrm{cell}\,\mathrm{types}}=30$ cell types, were normalized cell-wise to a target depth of 1,000 counts using Scanpy’s (version 1.11.1)^163^ ‘normalize_total’ function. Each spot k on tissue j comprised 30 cells, partitioned uniformly at random among the cell types present within the current region (${D}_{k}^{(j)}$). The integer vector containing the number of cells belonging to each type was normalized to produce a simplex serving as the true composition vector ${E}_{k}^{\,(j)}$. For each cell type t, $30{E}_{k,t}^{\,(j)}$ cells were drawn at random (without replacement) from ${G}^{(t)}$, the subset of cell profiles annotated as type t. Their expression profiles were summed to yield ${y}_{k,t}$ and then rounded to integer values to remove fractional reads introduced by ‘normalize_total’. As with the cluster-based approach, read counts were then pooled across all cell types, yielding spot-level reads ${y}_{k}={\sum }_{t}{y}_{k,t}$, and composition vectors were then (optionally) pooled within high-level annotation categories. Observational noise was added in an identical fashion to the cluster-based approach. For all cell-based data, cSplotch was run with an NB likelihood (without zero inflation) to account for overdispersion present over gene counts in the snRNA-seq profiles. As with the cluster-based approach, spots were assumed to be fully independent; thus, ${\psi }_{i}$ was suppressed in the GLM.

### Power sampling and its effect on estimation

ST data of distal colon sections from 12-week-old mice were chosen for subsampling analysis given the large number of samples from these mice (six mice (three males and three females) with 13, 8, 9, 8, 8 and 6 tissue sections per mouse). To subsample the data, a given number of mice were randomly selected and a given number of tissue sections were selected from each mouse (if a selected mouse had fewer than the given number of tissue sections, all tissue sections belonging to that mouse were taken). Each subsampled dataset (nine combinations of one, two and six mice with two, four and eight tissue sections per mouse) and the full dataset (52 tissue sections and six mice) was analyzed separately by cSplotch. To compare the estimated posterior distributions of *β*_*i*_ at the gene and tissue context levels, the posterior means and s.d. were calculated, which were then used to obtain normal distribution approximations of the posterior distributions. The KL divergence between the posteriors derived from the full and subsampled data (that is, how much information is lost when using the distribution estimated from the subsampled data) was used to quantify the differences between the normal distributions.

### Characterizing MCPs of gene expression

The cSplotch model output of posterior mean estimates of cellular gene expression $\{\bar{{\beta }_{1}},\ldots ,\bar{{\beta }_{k}\}}$ for k cell types in a given spatial niche (for example, the crypt apex MROI), where each ${\beta }_{i}$ is a matrix of dimensions ${N}_{\mathrm{genes}}\times {N}_{\mathrm{conditions}}$ that was used as input to identify gene sets spanning multiple cell types that show k-way correlation across the measured conditions. First, for each spatial niche, cell types and genes were selected for MCP analysis. Only cell types that are found in at least 5% of spatial spots, on average, were included, to focus on cell types with sufficient certainty in posterior estimates of gene expression. Across the k included cell types, the top 5% of gene signatures were considered on the basis of the coefficient of variation of $\bar{\beta }$ across the conditions to focus on genes with spatiotemporal variation.

Next, penalized matrix decomposition (PMD) was used to find linear combinations of gene signatures in each cell type that are maximally correlated across all conditions^164^. PMD seeks to find sparse canonical variates $\{{w}_{1},\ldots ,{w}_{k}\}$ that transform the original gene feature space into a new MCP feature space of dimension M<k:

15 $${\mathrm{argmax}}_{{w}_{1},\ldots ,{w}_{k}}\mathop{\sum }\limits_{i < j}{w}_{i}\bar{{\beta }_{i}}{\bar{\beta }}_{j}^{T}{w}_{j}$$

such that ${||}{w}_{i}{||}\le 1,{\rho }_{i}\left({w}_{i}\right) < {c}_{i}\forall i$, where ${\rho }_{i}({w}_{i})$ represent least absolute shrinkage and selection operator (LASSO) regression penalties and ${c}_{i}$ controls the degree of sparsity. These tuning parameters were chosen by R’s MultiCCA.permute function^108^. For each pair of cell types *i* and *j*,

16 $${\mathrm{argmax}}_{{w}_{i},{w}_{j}}{w}_{i}\bar{{\beta }_{i}}{\bar{\beta }}_{j}^{T}{w}_{j}={\mathrm{argmax}}_{{w}_{i},wj}\mathrm{corr}\left({\bar{\beta }}_{i}^{T}{w}_{i},{\bar{\beta }}_{j}^{T}{w}_{j}\right)$$

Therefore, the canonical covariates identified a space in which each MCP signal in cell type *i* is highly correlated with the corresponding signal in all other cell types. Next, given canonical variates from PMD, each of the M MCP signals was characterized by identifying at most *n* = 250 genes across all cell types that showed the strongest positive or negative contribution as measured by $\mathrm{abs}(w) > 0.1$. The list of correlated genes for each MCP, both ‘up’ genes with positive weight and ‘down’ genes with negative weight, was then used as input to a Fisher’s exact test to identify Kyoto Encyclopedia of Genes and Genomes (KEGG) functional gene sets enriched in the MCP. Each MROI was allowed at most k MCPs under the constraint that the bases for each MCP are orthogonal. As MCCA will always output programs in the same order, the maximum of k MCPs was calculated for a given spatial niche and latter programs that show low self-correlation among member genes (mean Pearson’s *r* < 0.3) or high cross-correlation with genes from other programs (mean Pearson’s *r* > 0.05) we optionally removed. Total MCP activity across conditions was calculated as a weighted sum of member gene activity (${y}_{m}={\sum }_{i}^{k}({\sum }_{g}^{G}({\bar{\,\beta }}_{i}[g,\bullet ]{w}_{i}[g,m])\,)$), whereas cellular contributions to each MCP were calculated by binning positive and negative weights by cellular origin.

### Spatiotemporal cellular composition analysis

To identify significant differences in cellular composition, each tissue section was treated as an independent sample and compositional estimates of all spots for a given MROI were pooled from the same section. Statistical significance across groups (for example, proximal–middle–distal samples) was assessed using Welch’s *t*-test. A crypt gradient gene was defined as one that showed a significant (BF > 2, log_2_ fold change > 0.5) difference in expression between crypt base and apex and a monotonic change in expression from base to base and mid, mid and apex.

### Reporting summary

Further information on research design is available in the Nature Portfolio Reporting Summary linked to this article.

## Online content

Any methods, additional references, Nature Portfolio reporting summaries, source data, extended data, supplementary information, acknowledgements, peer review information; details of author contributions and competing interests; and statements of data and code availability are available at 10.1038/s41587-025-02830-6.

## Supplementary information

Supplementary Information Extended Data Figs. 11–14 and Supplementary Methods.

Reporting Summary

Supplementary Tables 1–6 Supplementary Table 1: Composition of ST dataset by covariate group and spot annotation. Supplementary Table 2: Composition of snRNA-seq dataset by cell type. Supplementary Table 3: Semantic segmentation model and morphological superclass specifications. Supplementary Table 4: Cell type marker genes for deconvolution. Supplementary Table 5: Simulated ST data specifications. Supplementary Table 6: Genes exhibiting sex-based DE in at least one sample condition.

Supplementary Table 7 Regional variation in mean cell composition and gene expression.

Supplementary Table 8 Crypt gradient genes.

Supplementary Table 9 Cellular associations of crypt gradient genes.

Supplementary Table 10 Significant variations in cell composition across time.

Supplementary Table 11 Genes and functional annotation categories showing temporal DE across aging windows in colon crypt.

Supplementary Table 12 Cellular composition and activity of all MCPs along the vertical crypt axis during colon aging.

Supplementary Table 13 Manually curated functional annotation of selected MCPs along the vertical crypt axis during colon aging.

Supplementary Table 14 Significant associations between crypt MCPs and KEGG pathways.

## Data Availability

All processed data were deposited to the Single Cell Portal under accession number SCP2595. All raw data were deposited to the National Center for Biotechnology Information Gene Expression Omnibus repository under accession numbers GSE285985 and GSE284137.

## Code availability

All code is deposited on GitHub (https://github.com/adaly/cSplotch). A Google Cloud-enabled workflow and data exploration tool are available on Terra Firecloud (https://app.terra.bio/#workspaces/techinno/cSplotch%20Workflow).
