# Supplementary material for: Tissue and cellular spatiotemporal dynamics in colon aging
Source: Nat Biotechnol. 2025 Oct 22;44(8):1340–52. doi: 10.1038/s41587-025-02830-6 (PMC13461361; doi:10.1038/s41587-025-02830-6)
Supplement: Supplementary file 1 [file 41587_2025_2830_MOESM1_ESM.pdf]

nature biotechnology

Supplementary information

https://doi.org/10.1038/s41587-025-02830-6

Tissue and cellular spatiotemporal dynamics in colon aging

In the format provided by the authors and unedited

Table of Contents

Extended Data Figure 11: Enhanced cell marker specificity after integration of snRNA-seq and ST. **2**

Extended Data Figure 12: Gene pathways demonstrating spatial gradient behavior across the colon crypt axis. **3**

Extended Data Figure 13: Spatio-temporal variation in inferred cellular composition in the crypt. **5**

Extended Data Figure 14: Correlation among member genes for MCPs derived in colon crypt MROIs. **6**

Supplementary Methods **7**

- *Murine tissue collections* 7
- *Immunostaining and epifluorescent microscopy* 8
- *Histology for Spatial Transcriptomics* 10
- *In situ and library preparation of Spatial Transcriptomics reactions* 10
- *Nucleus extraction and library preparation for snRNA-seq* 13
- *Spatial Transcriptomics sequencing and demultiplexing* 13
- *snRNA-seq sequencing and demultiplexing* 14
- *Analysis of snRNA-seq colon data* 14
- *Gene set enrichment analysis* 17

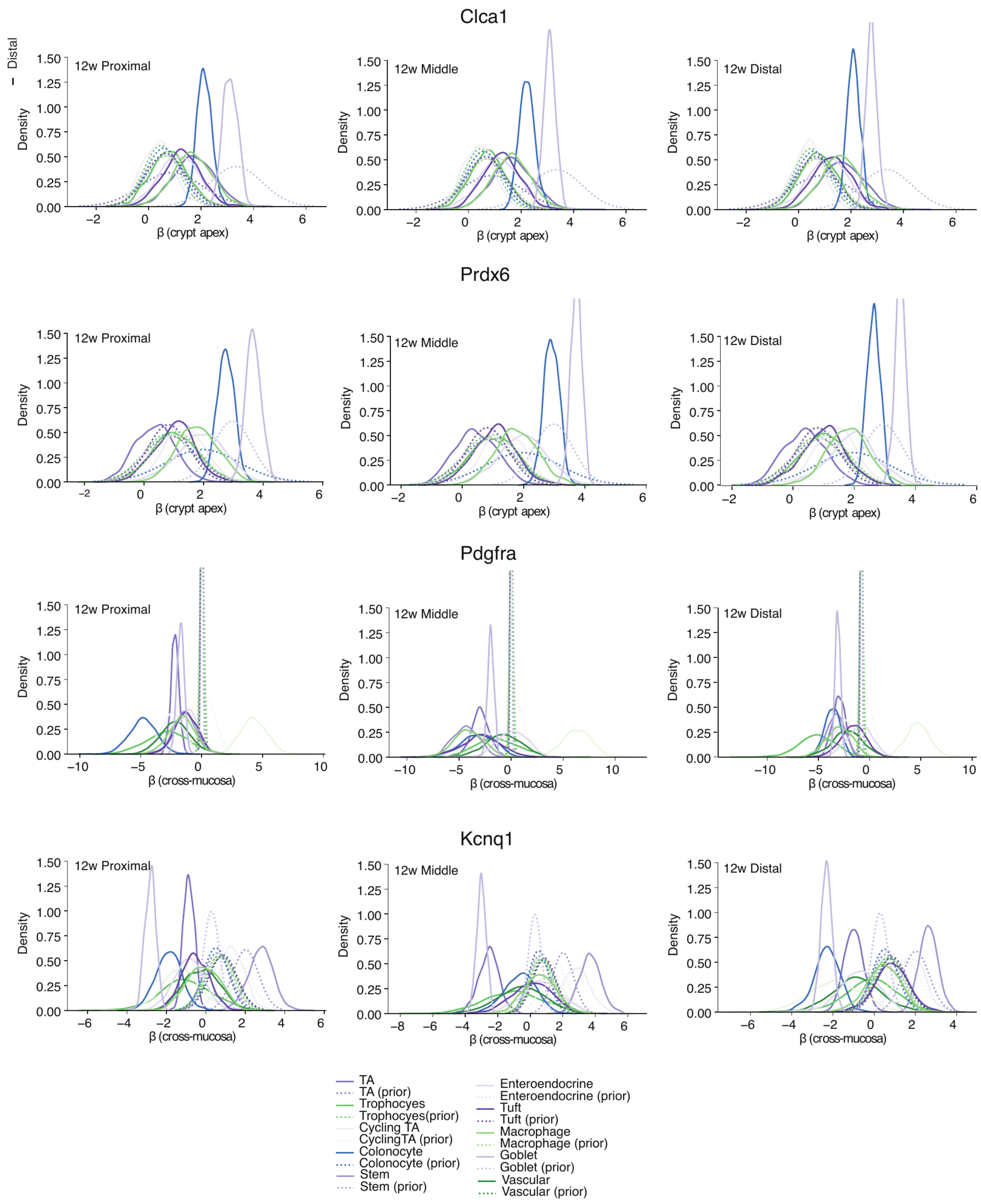

Extended Data Figure 11: Enhanced cell marker specificity after integration of snRNA-seq and ST. Distribution of characteristic expression rate (β; x-axis) for canonical marker genes of select cell types (color; *Clca1*, *Prdx6* (goblet cells); *Pdgfra* (fibroblasts), and *Kcnq1*(TA cells)) in specific MROIs (x-axis label) from proximal (left), middle (middle) and distal (right) colon segments from 12 week old mice based on snRNA-seq data only (dashed lines; empirical prior), or as inferred by cSplotch from ST and snRNA-seq data (solid lines; posterior distributions). Only cell types present at least at 2% in MROI-annotated ST spots are included.

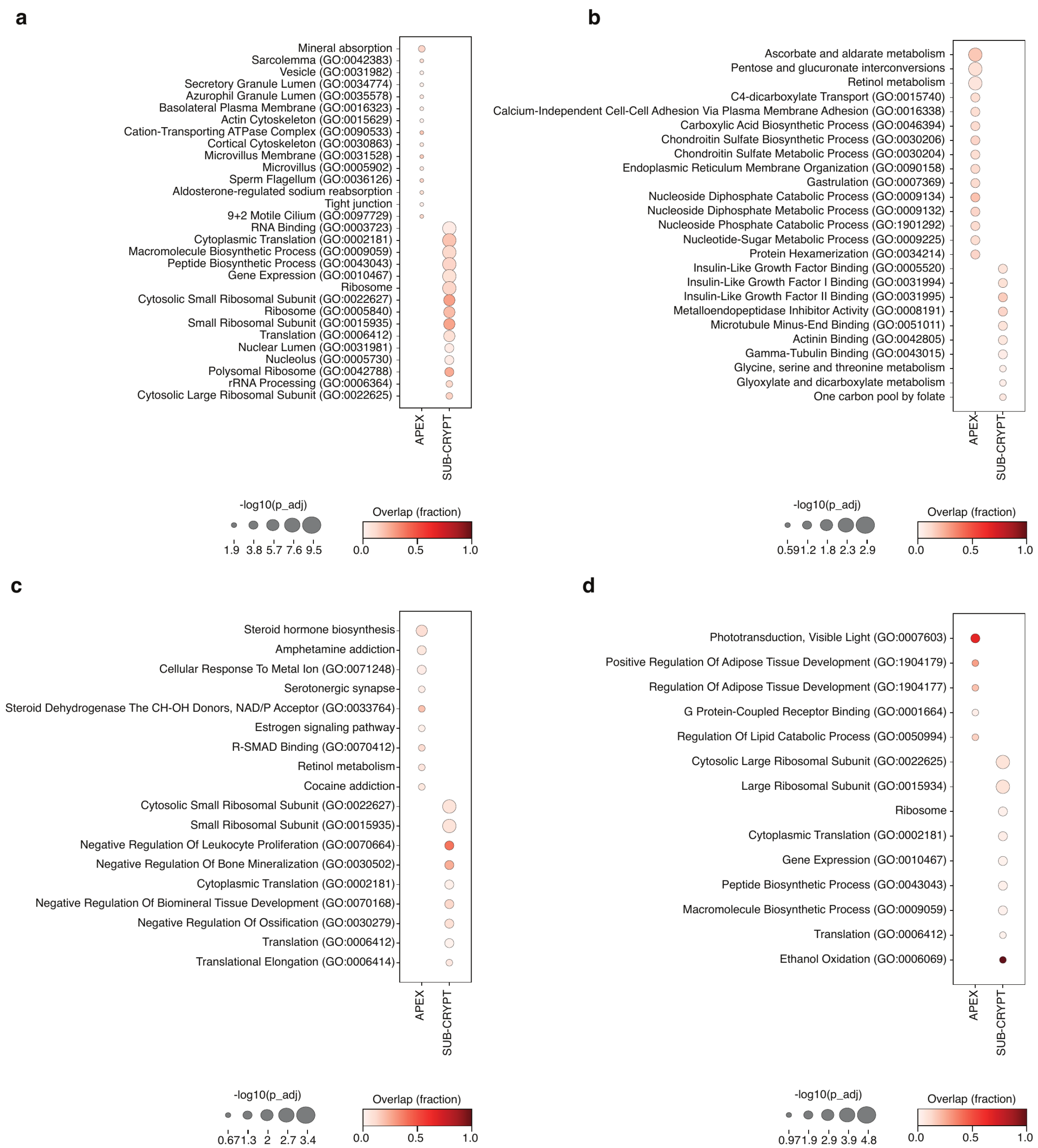

Extended Data Figure 12: Gene pathways demonstrating spatial gradient behavior across the colon crypt axis. GSEA analysis for genes marked by cSplotch as showing gradient expression across the crypt (DE and monotonic expression change between APEX and SUB-CRYPT) in (**a**) all colon regions (n=321 genes), (**b**) proximal colon exclusively (n=23 genes), (**c**) middle colon exclusively (n=53 genes), and (**d**) distal colon exclusively (n=42 genes). Dot size: -log10(p val). Color scale: Overlap between regional DE genes and GSEA process.

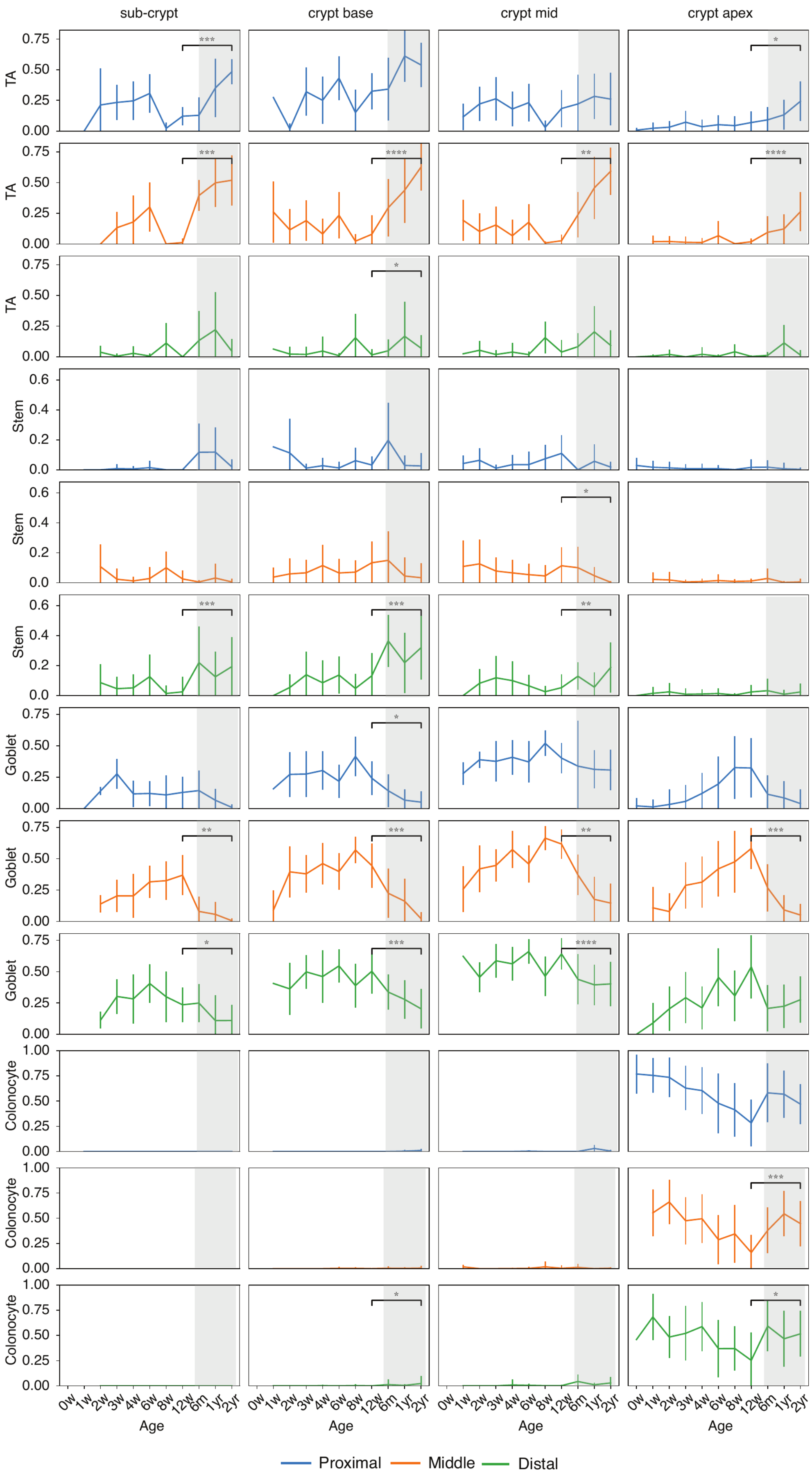

sub-crypt
crypt base
crypt mid
crypt apex
TA
Stem
Goblet
Colonocyte
0w
1w
2w
3w
4w
6w
8w
12w
6m
1yr
2yr
Age
Proximal
Middle
Distal

Extended Data Figure 13: Spatio-temporal variation in inferred cellular composition in the crypt. Fraction of cells (y axis; mean and standard deviation) of four abundant (>5% of spots of average) cell type (rows) at each time point (x axis) in each of four crypt MROIs (columns) in each colon region (color code). Gray area: aging window; brackets: significant changes between 12w and 2yr time points; *: 0.01 < FDR <= 0.05; **: 10^{-3} < FDR <= 0.01; ***: 10^{-4} < FDR <= 10^{-3}; ****: FDR <= 10^{-4} (two-sided Welch's *t*-test). Significant changes are also listed in **Extended Data Table 10**.

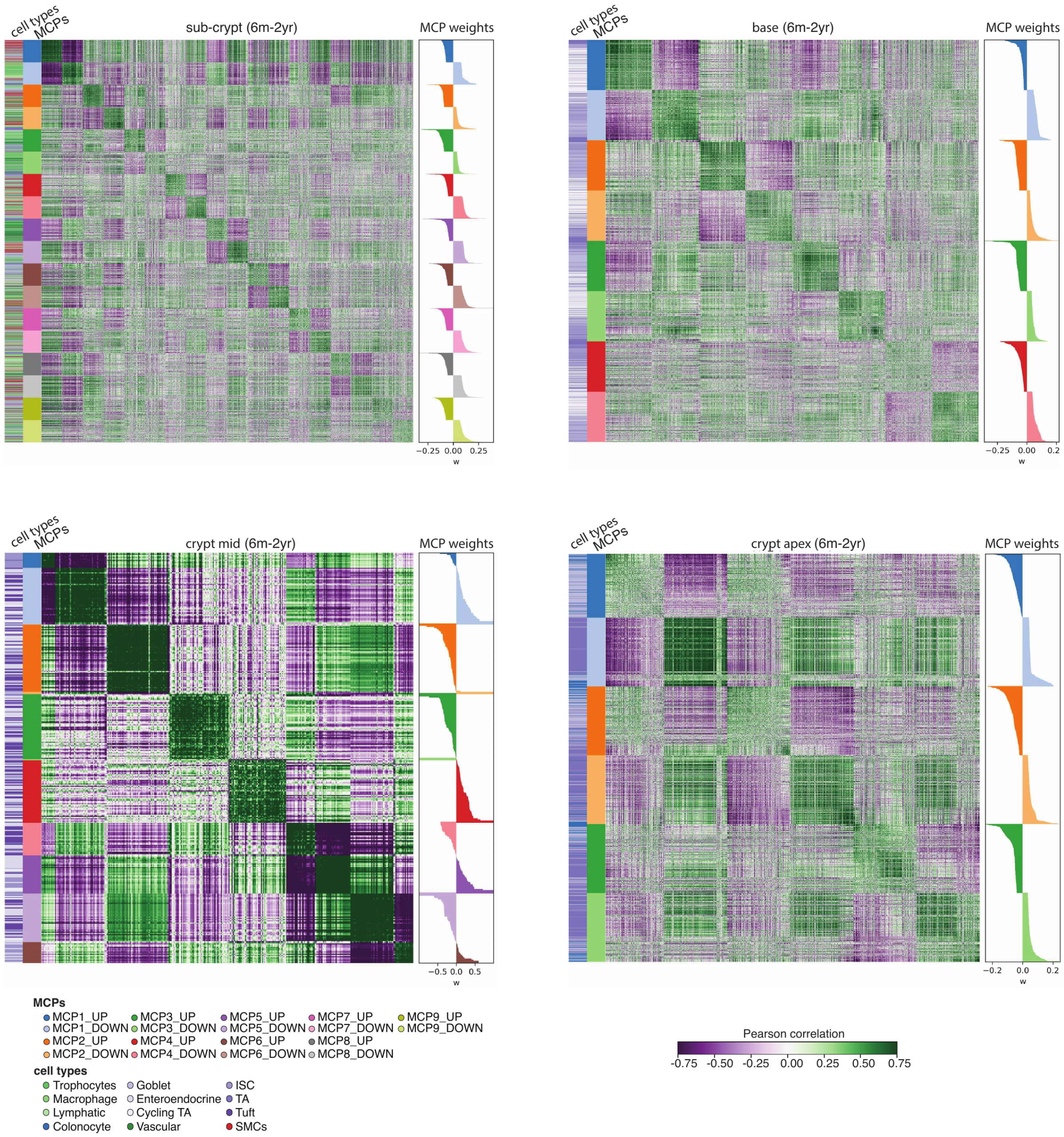

Extended Data Figure 14: Correlation among member genes for MCPs derived in colon crypt MROIs. Heatmaps demonstrate correlation between inferred cellular gene expression signatures (cell type + gene; x and y axes) across sample covariates (age and colon region), with rows/columns clustered by MCP assignment (color code; left) and displaying cell type of origin (color code; far left). Barplots (right) denote weight (w; x-axis) given to each gene signature within its assigned MCP (shared color code). MCPs are independently derived in each of the four MROIs indicated in figure supertitles.

Supplementary Methods

Murine tissue collections

C57BL/6J mice were obtained from The Jackson Laboratory (Bar Harbor, ME) and maintained in accordance with ethical guidelines monitored by the Institutional Animal Care and Use Committees (IACUC) established by the Division of Comparative Medicine at the Broad Institute of MIT and Harvard and Columbia University, and consistent with the Guide for Care and Use of Laboratory Animals, National Research Council, 1996 (institutional animal welfare assurance no. A4711-01), with protocols 0122-10-16 and AABI3617, respectively. All mice were maintained under SPF conditions on a 12-h light–dark cycle at an ambient temperature of 21.5 ± 1 °C with relative humidity between 30% and 70% and were provided food and water ad libitum. Colons were collected within 5 min of death and flushed using ice-cold 1x PBS (Gibco) using a straight gauge needle with a 2.4mm tip (Kent Scientific Corporation, USA) for mice >3 weeks of age or a newborn feeding needle (Cadence Science, USA) for mice ages 3 weeks. The rest of the mice were processed without colon flushing prior to freezing. Tissues were then dried and embedded in Optimal Cutting Temperature (OCT, Fisher Healthcare, USA) in large molds (VWR, USA). Samples were then plunged onto a metal plate pre-chilled and sitting on top of dry ice for 2min or until complete freezing. Samples were transferred and stored at -80°C until use. Cryosections were cut at 10µm thickness onto ST slides, and stored at -80°C for at most 2 days. For nucleus extractions, colons were separated from the animals within 5 minutes of death and each colon was separately flushed similar to collecting tissue for ST. Approximately 0.5cm of tissue from three different colon regions was placed on a tissue and dried before transfer to a sterile dish to ensure all excess water was gently removed from the sample. Tissues were then placed in a 1.5 mL tube on dry ice and, upon freezing, transferred to −80°C until subsequent tissue processing.

Immunostaining and epifluorescent microscopy

A superfrost slide (ThermoFisher Scientific, USA) with a mounted tissue section was dried to 37°C for 4 minutes on a thermal incubator (Eppendorf Thermomixer Option C, Germany) followed by *in situ* fixation in 4% PFA (Sigma Aldrich, USA) at RT and washing in 50mL 1x PBS (Gibco). Slides were placed in a chamber holder (ProPlate Multi-Array slide system; GraceBioLabs, USA) to allow for incubations in predefined conditions and volumes. All following antibody incubations were performed at 4°C. To block tissues from nonspecific antibody binding, 1:100 TruStain FcX™ PLUS (anti-mouse CD16/32, Biolegend, USA) antibody in 1x Perm/Wash buffer (BD, USA) was added and tissues were incubated for 30min. Tissues were washed 3 times with 1x PBS-T (0.05% Tween-20, Sigma, USA). Antibodies were added at a 1:100 dilution in 1x Perm/Wash buffer and incubated for 30min. If an unlabeled primary antibody was used, tissues were again washed and stained with a labeled secondary antibody prepared in 1x Perm/Wash buffer (BD, USA). Tissues were then washed in the same fashion before counterstaining with Hoechst (10mg/ml, ThermoFisher Scientific, USA) diluted 1:1000 in 1xPBS (ThermoFisher Scientific, USA) for 5 min. This was followed by another wash cycle after which slides were air dried and mounted with 85% glycerol prior to imaging. Primary antibodies were diluted and clone and provider information were as follows: EPCAM (Biolegend, Alexa-647 labeled primary, clone G8.8, 1:100 dilution), TFF3 (Abcam, unlabeled primary, EPR26048-14, 1:100 dilution), SMA (Abcam, unlabeled primary, clone EPR5368, 1:100 dilution), FABP4 (Abcam, Alexa-647 labeled, clone EPR3579, 1:250 dilution), FABP7 (Abcam, unlabeled primary, clone EPR24033-13, 1:100 dilution), CD48 (Biolegend, APC labeled, clone HM48-1, 1:100 dilution), PRDX6 (Abcam unlabeled primary antibody, clone EPR3754, 1:500 dilution), CD74 (Biolegend unlabeled primary

antibody, clone In1/CD74, 1:250 dilution), PDGRFA (Abcam unlabeled primary antibody, clone EPR22059-270, 1:1000 dilution), goat anti-rat IgG (Life Technologies, Alexa Fluor 488 Goat anti-rat IgG, 1:100 dilution), donkey anti-rabbit IgG (Biolegend, Alexa 647 labeled, clone Poly6064, 1:100 dilution). Epifluorescent images were acquired on an Axio Imager Z2 microscope using a PhotoFLuor LM-75 lightsource (89North, USA) in combination with a Plan-APOCHROMAT 40x/0.75 objective (Carl Zeiss, Germany). Images were stitched using Vslide (v1.0.0, MetaSystems GmbH). Prior to cross-image quantitative IF comparisons, histogram matching was performed using scikit-image[1] and signals were thresholded against the mean intensity around the image border.

Slide production

Spatially barcoded arrays were printed on Codelink glass slides with 200um center-to-center distance using the ArrayJet Spider system (ArrayJet LTD, Scotland, UK), with six active surfaces per slide[2,3]. Using Codelink chemistry (Surmodics, USA), 5' amine-modified DNA oligonucleotides (5'-[AmC6]dUdUdUdUd-[Illumina_adaptor]-[spatial barcode]-[UMI]-[20T]-VN) (IDT, USA) were bound to the slide surface using 100pL droplet deposition (ArrayJet LTD, Scotland, UK), This patterning formed 100µm spots with a 200µm spot-to-spot pitch distances, for 1,007 spatially barcoded spot conjugations in a 6.2mm x 6.6mm capture area. Finally, the unconjugated surface was blocked using a blocking buffer at 50°C (50 mM ethanolamine, 0.1 M Tris, pH 9) for 30min before washing the slides in 4x SSC, 0.1% SDS (pre-warmed to 50°C) for 30 min, rinsing them with deionized water and drying.

Histology for Spatial Transcriptomics

Tissue sections were adhered to the ST arrays at 37°C for 1 min, *in situ* fixated in 4% PFA (Sigma Aldrich, USA) at RT and washed in 50mL 1x PBS (Gibco). In most cases, 4 tissue sections were fitted onto one ST active area. Tissues were then dried for 1min in isopropanol, followed by hematoxylin and eosin (H&E) staining. Briefly, tissue sections were exposed to 100% Mayer's hematoxylin (DAKO, Agilent) for 6 minutes followed by washing for 2 min in deionized water at RT. To adjust the pH, slides were briefly dipped in DAKO's Bluing buffer (Agilent) and then counterstained in 5% eosin diluted in Tris-AA (pH 7.2) for 1 min. Slides were again washed in deionized water and dried prior to mounting them with 85% glycerol prior to imaging. All samples were imaged on an Axio Imager Z2 microscope equipped with a 20x/0.8 Plan-APOCHROMAT (Carl Zeiss Microscopy, Germany) and the resulting images stitched with Vslide (v1.0.0, MetaSystems GmbH).

In situ and library preparation of Spatial Transcriptomics reactions

After imaging, coverslips were removed in deionized water and permeabilization reactions immediately started. First, tissue samples were permeabilized with 120µl reagent per reaction of collagenase I (200U) in 1x HBSS (both from ThermoFisher Scientific, USA). Pre-permeabilization reagent removal was followed by a 180µl wash in 0.1X Saline Sodium Citrate (SSC, Sigma-Aldrich, USA) at 37°C. Next, tissue was permeabilized with 75µl 0.1% pepsin (pH 1, Sigma-Aldrich, USA) at 37°C for 10min, followed by another wash with 0.1x SCC. Reverse transcription (RT) was performed by the addition of 75µl RT reagents: 50ng/µl actinomycin D (Sigma-Aldrich, USA), 0.5mM dNTPs (ThermoFisher Scientific, USA), 0.20µg/µl BSA, 1X First strand buffer, 5mM DTT, 2U/µl RNaseOUT, 20U/µl Superscript III (all from ThermoFisher Scientific, USA).

Tissues were digested from the slide surface by 1h incubation in proteinase K (Qiagen, Germany) at 56°C. Slides were washed as suggested by the slide manufactures (Codelink, Surmodics): 10min 2x SCC with 0.1% SDS (Sigma Aldrich), 1min 0.2x SCC and 1min 0.1X SSC. To remove spatial cDNA:mRNA hybrids from the array surface, Uracil-Specific Excision Reagent (NEB, USA) was used[4]. The reaction was run for 2h at 37°C and the resulting spatially barcoded cDNA libraries were collected and libraries prepared[4]. Briefly, cDNA:RNA hybrids collected from the array surface were used as input in the first part of library preparation reactions. RNA strands were digested and used as primer to make dsDNA using DNA Polymerase I and RNaseH (2.7X First strand buffer, 3.7 U/µl DNA polymerase I and 0.2 U/µl Ribonuclease H (all from ThermoFisher Scientific, USA)) for 2h at 16°C. The material was made into blunt-end dsRNA products with 15U T4 DNA polymerase (NEB, USA) for 20 minutes at 16°C and reactions stopped by addition of 20mM EDTA (pH 8.0, ThermoFisher Scientific, USA). dsDNA was purified using Ampure XP (Beckman Coulter, USA) at a bead to cDNA ratio of 1:1. Next, the material was linearly amplified using a T7 promoter sequencing initially embedding in the oligonucleotides on the array surface by adding 27.8µl of the T7 reaction mix (46.2mM rNTPs, 1.5X T7 reaction buffer, 1.54 U/µl SUPERaseIN inhibitor and 2.3U/µl T7 enzyme; all from ThermoFisher Scientific, USA) for 14h at 37°C. This was followed by a bead cleanup with RNAclean Ampure XP beads (Beckman Coulter, USA) at a beads:aRNA ratio of 1.8:1. 8µl of the eluted aRNA was used as input to the following reactions. 2.5µl of 3µM aRNA adapters [rApp]AGATCGGAAGAGCACACGTCTGAACTCCAGTCAC[ddC] were added to 8µl of aRNA. The reaction was then incubated at 70°C in a PCR machine for 2min and immediately chilled on wet ice. To ligate the adaptors, 4.5µl T4 RNA ligation mix (3.3X T4 RNA ligase buffer, 66U/µl truncated T4 ligase 2 and 13U/µl murine RNAse inhibitor (all from NEB, USA)) were

added at 25°C for 1h, followed by an Ampure XP (Beckam Coulter, USA) bead purification at a bead:cDNA ratio of 1.8:1. 1:1 v/v of 20µM GTGACTGGAGTTCAGACGTGTGCTCTTCCGA and 10mM dNTPs were added to the ligated samples and heated to 65°C for 5min. Reverse transcription took place by adding 2.5X First strand buffer, 13mM DTT, 5 U/µl RNaseOUT and 25 U/µl Superscript III (all from Thermo Fisher Scientific, USA). Samples were incubated at 50°C for 1h. 10µl of nuclease-free water were added followed by a final Ampure XP bead purification at bead:cDNA ratio of 1.7:1 with a final elution of 10µl nuclease-free water. qPCR was performed to determine the optimal number of cycles for PCR indexing[4]. For each reaction, a 60.1 µL qPCR mix (final concentrations: 1.25× KAPA mix, 0.63 µM InPE 1.0, 0.0125 µM InPE 2.0, 0.063 µM Illumina PCR index primer, 1.3× EVA Green) was prepared on ice, and 8 µL was dispensed into each of six qPCR wells. PCR primer sequences followed the form CAAGCAGAAGACGGCATACGAGATXXXXXXGTGACTGGAGTTC, with XXXXXX replaced with one of 12 possible values (CGTGAT, ACATCG, GCCTAA, TGGTCA, CACTGT, ATTGGC, GATCTG, TCAAGT, CTGATC, AAGCTA, GTAGCC, or TACAAG) chosen independently at random for each sample. Two microliters of each sample were added, mixed, centrifuged (~1,000 g, 30 s, RT), and amplified (98 °C, 3 min; 2–25 cycles of 98 °C, 20 s; 60 °C, 30 s; 72 °C, 30 s) with a heated lid (105 °C). The optimal cycle number for indexing was determined as the cycle at maximum fluorescence minus 3–4 cycles[4]. PCR indexing reactions (40 µL total volume; final concentrations as above with 25µL KAPA mix, 1µL PCR InPE mix, 1µL PCR index) were prepared, mixed with 5 µL of sample, centrifuged (2,000 g, 5 s, RT), and amplified (98 °C, 3 min; N cycles of 98 °C, 20 s; 60 °C, 30 s; 72 °C, 30 s; final extension 72 °C, 5 min; hold at 4 °C). Twenty-five microliters of nuclease-free water were added to each PCR product.

Nucleus extraction and library preparation for snRNA-seq

SnRNA-Seq was performed on frozen tissues taken from 1.5ml tubes used for storage at −80°C and placed in pre-chilled 1 mL extraction buffer (0.03% Tween-20, 146mM NaCl, 10mM Tris pH 8.0, 1mMM CaCl2 and 21mM MgCl2; all from Sigma Aldrich, USA), supplemented with 400U RNasin Plus inhibitor (Promega Corporation, USA) and 200U SUPERaseIn RNase Inhibitor (ThermoFisher Scientific, USA). Tissues were disintegrated by chopping with tungsten Carbide Straight 11.5 cm Fine Scissors (14558-11, Fine Science Tools, Foster City, CA) for 10 minutes on ice. To avoid clogging, samples were filled through a 40 µm strainer (Falcon). To release any leftover material from the strainer, it was cleaned with the extraction buffer without the addition of Tween-20, followed by centrifugation to pellet the nuclei at 500g for 5 mins at 4°C. Supernatants were removed and nuclei were resuspended in a 100µL extraction buffer without the addition of Tween-20 before filtering through a 40 µm strainer-capped into a round bottom tube (Falcon). Nuclei were counted and ~8,000 nuclei were loaded per channel on the GemCode Single Cell Platform using the GemCode Gel Bead kit, Chip and Library Kits (10X Genomics, Pleasanton, CA), following the manufacturer's protocol. Briefly, nuclei were partitioned into Gel Beads in Emulsion (GEMs), lysed and barcoded using reverse transcription reactions, followed by amplification, shearing and 5′ adapter and library indexing[5,6].

Spatial Transcriptomics sequencing and demultiplexing

ST cDNA libraries were diluted to 4nM and 1.08pm libraries loaded for sequencing on an Illumina NextSeq 550 (Illumina, USA) using paired-end sequencing (R1 30bp, R2 55bp). Samples were sequenced at a mean depth of 65.6 million paired-end reads depth. fastq reads were generated with

bcl2fastq2. ST Pipeline[7] was used to process the resulting fastq files. Briefly, 5nt trimmed R2 was used for mapping to the mouse genome using STAR[8]. After that, mapped reads were annotated using HTseq-count[9]. Spatial barcodes were collapsed using TagGD[7,10] modified demultiplexer (k-mer 6, mismatches 2). Then, unique molecular identifiers (UMIs) mapped to the same transcript and spatial barcode were collapsed using naive clustering with one mismatch allowed[11]. The output genes-by-barcode matrix was used in all further processing steps. Average library saturation was 78.3%. To focus on reliably detected genes across spots, genes detected in fewer than 2% of spots were removed, as were spots with fewer than 100 UMIs across all genes. The resulting median number of genes and UMI transcripts per spatial spot was 2,164 (10th percentile was 829 and 90th percentile was 4,370) and 4,343 (10th percentile was 1,246 and 90th percentile was 13,055).

snRNA-seq sequencing and demultiplexing

Libraries were sequenced on an Illumina NextSeq 550 (R1: 26 bases; R2: 55 bases) or a NovaSeq 6000 (R1: 28 bases; R2: 94 bases). CellRanger v3.0 was used for all initial data pre-processing. Fastq reads were first demultiplexed and then mapped to the reference mm10 transcriptome, augmented to allow for counting of all transcript tags in addition to counting exons as suggested by 10X Genomics. Each barcode was connected to a particular cell and UMIs were collapsed to account for duplicated transcripts. Filtered matrices reflecting digital gene expression (DGE) for each sample and cell were extracted from the pipeline.

Analysis of snRNA-seq colon data

DGE matrices were concatenated from samples collected at 10 different ages. Potential doublets were removed using scrublet[12] (~12% of barcodes in the dataset). To facilitate downstream

analyses, all snRNA-seq data were combined with the mouse colon droplet data from Drokhlyansky *et al*[6] (also collected by our lab). Nucleus profiles with at least 800 genes expressed in a minimum of 10 cells were kept for further analysis. To ensure that only highest quality profiles were retained, profiles with less than 800 UMIs or more than 30% mitochondrial or ribosomal transcripts were also removed. Data were then normalized by the total number of transcripts or UMIs per nucleus profile and converted to transcripts-per-10,000 to account for differences in sequencing depth. Mean (μ) and coefficient of variation (CV) of expression were calculated for each gene in the dataset and partitioned into 20 equal-frequency bins. LOESS regression was used to fit the relationship between log(CV) and log(μ). Genes with the 1,500 highest residuals were equally sampled across these bins. To account for differences in batches, this was performed for each sample separately and a consensus list of 1,500 genes with the highest recovery rates was selected, with ties broken by random sampling. Genes were considered as differentially expressed only if $\log_2$(fold change)>1 and Benjamini-Hochberg (BH) FDR<0.01 (likelihood ratio test). Additionally, to account for cycling cells, both cell cycle scores (as in scanpy.tl.score_genes_cell_cycle[13]) and mitochondrial content scores in each cell were regressed prior to data clustering. All of the following processing steps including clustering were performed with scanpy (v1.11.1)[13]. Overall, final analyzed data included 403,797 nucleus profiles with an average 2,281 genes and 4,305 UMIs per nucleus.

To avoid clustering cell profiles based on substantial variability between different mice and ages, which is likely a combination of technical and biological differences, Harmony[14] was used for batch correction as follows. Dimensionality reduction was performed using principal components analysis (PCA) with batches denoted as "mouse sample identifiers", which reflected the animal identifier, and then a *k*-nearest neighbors (*k*-NN) was constructed using estimated k=20 neighbors

and the first 40 PCs. Convergence was reached after 10 iterations and the Harmony corrected reduced data was then clustered using Phenograph[15] with *k*=25 nearest neighbors using the Minkowski metric. After clustering, cell type labels from Drokhlyansky *et al*[6] were manually transferred to annotate clusters. Cell type labels were transferred to three external datasets[16–18] by an identical methodology to facilitate comparison of inferred cSplotch transcriptomes to these held-out data.

Visium and cSplotch sample processing

Visium spatial gene expression data were generated following the guidelines provided in the Visium Spatial Gene Expression User Guide (10x Genomics, CG000239 Rev F). Briefly, 10 µm proximal colon tissue sections were placed on Visium Spatial Gene Expression Slides (10x Genomics, catalog no. 1000185) and fixed in ice-cold methanol at -20°C for 30 minutes, followed by histological staining with H&E as described above. Each processed Visium array contained sections for a different animal. Brightfield images were captured using a 10x Plan-Apochromat 10x/0.45 M27 objective on a Zeiss Axio Imager Z2 equipped with a CoolCube 4 camera (MetaSystems, Germany). Tissue sections underwent a 10-minute permeabilization with 0.1% pepsin (Sigma-Aldrich, USA) in 0.1M HCl at 37°C, optimized based on tissue permeabilization time-course experiments. Reverse transcription was then performed to generate cDNA molecules, incorporating spatial barcodes and unique molecular identifiers (UMIs) according to the Visium protocol. Following second strand synthesis, cDNA was collected and amplified, with the optimal number of amplification cycles for each Visium array determined using qPCR, as specified in the Visium Spatial Gene Expression User Guide (10x Genomics, CG000239 Rev F). Visium libraries were prepared following the Visium Spatial Gene Expression User Guide. To ensure compatibility

with Illumina sequencing, specific PCR primers were incorporated during library preparation (Dual Index Kit TT Set A, 10x Genomics). The total yield of the prepared libraries was measured using a Qubit 2.0 fluorometer and the distribution profiles of the final libraries were evaluated with the 2100 Bioanalyzer system (Agilent, USA). The libraries were loaded on the NextSeq 500/550 High-Output v2.5 Kit (150 cycles) for paired-end sequencing (28 bp forwards and 100 bp reverse read) (Illumina, USA). FASTQ files were processed with 10x Genomics Spaceranger count software to produce count matrices using the default murine reference transcriptome. Visium spots with fewer than 1,000 UMIs were discarded prior to cSplotch analysis due to low signal, after which point the median sequencing depth across all spots was 8,299 UMIs. cSplotch was run on Visium data as a two-level model (level 1: age + colon region, level 2: mouse ID), and HMC sampling was run with four chains per gene with 250 samples per chain.

Gene set enrichment analysis

Gene set enrichment analysis was carried out using GSEApy's (v1.1.3)[19] "enrichr" function. Four gene sets were considered for all analyses: 'GO_Cellular_Component_2023', 'GO_Molecular_Function_2023', 'GO_Biological_Process_2023', and 'KEGG_2019_Mouse', and the set of all gene processed with cSplotch was used as the background signal.

References

1. van der Walt, S. *et al.* scikit-image: image processing in Python. *PeerJ* **2**, e453 (2014).
2. Vickovic, S. *et al.* Massive and parallel expression profiling using microarrayed single-cell sequencing. *Nat. Commun.* **7**, 13182 (2016).

3. Ståhl, P. L. *et al.* Visualization and analysis of gene expression in tissue sections by spatial transcriptomics. *Science* **353**, 78–82 (2016).
4. Salmén, F. *et al.* Barcoded solid-phase RNA capture for Spatial Transcriptomics profiling in mammalian tissue sections. *Nat. Protoc.* **13**, 2501–2534 (2018).
5. Slyper, M. *et al.* A single-cell and single-nucleus RNA-Seq toolbox for fresh and frozen human tumors. *Nat. Med.* **26**, 792–802 (2020).
6. Drokhlyansky, E. *et al.* The Human and Mouse Enteric Nervous System at Single-Cell Resolution. *Cell* **182**, 1606-1622.e23 (2020).
7. Navarro, J. F., Sjöstrand, J., Salmén, F., Lundeberg, J. & Ståhl, P. L. ST Pipeline: an automated pipeline for spatial mapping of unique transcripts. *Bioinformatics* **33**, 2591–2593 (2017).
8. Dobin, A. *et al.* STAR: ultrafast universal RNA-seq aligner. *Bioinformatics* **29**, 15–21 (2013).
9. Anders, S., Pyl, P. T. & Huber, W. HTSeq--a Python framework to work with high-throughput sequencing data. *Bioinformatics* **31**, 166–169 (2015).
10. Costea, P. I., Lundeberg, J. & Akan, P. TagGD: fast and accurate software for DNA Tag generation and demultiplexing. *PLoS One* **8**, e57521 (2013).
11. Smith, T., Heger, A. & Sudbery, I. UMI-tools: modeling sequencing errors in Unique Molecular Identifiers to improve quantification accuracy. *Genome Res.* **27**, 491–499 (2017).
12. Wolock, S. L., Lopez, R. & Klein, A. M. Scrublet: Computational Identification of Cell Doublets in Single-Cell Transcriptomic Data. *Cell Syst* **8**, 281-291.e9 (2019).
13. Wolf, F. A., Angerer, P. & Theis, F. J. SCANPY: large-scale single-cell gene expression data analysis. *Genome Biol.* **19**, 15 (2018).

14. Korsunsky, I. *et al.* Fast, sensitive and accurate integration of single-cell data with Harmony. *Nat. Methods* **16**, 1289–1296 (2019).

15. Levine, J. H. *et al.* Data-Driven Phenotypic Dissection of AML Reveals Progenitor-like Cells that Correlate with Prognosis. *Cell* **162**, 184–197 (2015).

16. Nyström, E. E. L. *et al.* An intercrypt subpopulation of goblet cells is essential for colonic mucus barrier function. *Science* **372**, (2021).

17. Jasso, G. J. *et al.* Colon stroma mediates an inflammation-driven fibroblastic response controlling matrix remodeling and healing. *PLoS Biol.* **20**, e3001532 (2022).

18. Širvinskas, D. *et al.* Single-cell atlas of the aging mouse colon. *iScience* **25**, 104202 (2022).

19. Fang, Z., Liu, X. & Peltz, G. GSEApy: a comprehensive package for performing gene set enrichment analysis in Python. *Bioinformatics* **39**, (2023).
