# Supplementary material for: Tissue and cellular spatiotemporal dynamics in colon aging
Source: Nat Biotechnol. 2025 Oct 22;44(8):1340–52. doi: 10.1038/s41587-025-02830-6 (PMC13461361; doi:10.1038/s41587-025-02830-6)
Supplement: Supplementary file 2 [file 41587_2025_2830_MOESM2_ESM.pdf]

nature portfolio

Corresponding author(s): Sanja Vickovic

Last updated by author(s): Jul 21, 2025

Reporting Summary

Nature Portfolio wishes to improve the reproducibility of the work that we publish. This form provides structure for consistency and transparency in reporting. For further information on Nature Portfolio policies, see our Editorial Policies and the Editorial Policy Checklist.

Statistics

For all statistical analyses, confirm that the following items are present in the figure legend, table legend, main text, or Methods section.

n/a	Confirmed	
☐	☒	The exact sample size (*n*) for each experimental group/condition, given as a discrete number and unit of measurement
☐	☒	A statement on whether measurements were taken from distinct samples or whether the same sample was measured repeatedly
☐	☒	The statistical test(s) used AND whether they are one- or two-sided<br>*Only common tests should be described solely by name; describe more complex techniques in the Methods section.*
☐	☒	A description of all covariates tested
☐	☒	A description of any assumptions or corrections, such as tests of normality and adjustment for multiple comparisons
☐	☒	A full description of the statistical parameters including central tendency (e.g. means) or other basic estimates (e.g. regression coefficient) AND variation (e.g. standard deviation) or associated estimates of uncertainty (e.g. confidence intervals)
☐	☒	For null hypothesis testing, the test statistic (e.g. *F*, *t*, *r*) with confidence intervals, effect sizes, degrees of freedom and *P* value noted<br>*Give P values as exact values whenever suitable.*
☐	☒	For Bayesian analysis, information on the choice of priors and Markov chain Monte Carlo settings
☐	☒	For hierarchical and complex designs, identification of the appropriate level for tests and full reporting of outcomes
☐	☒	Estimates of effect sizes (e.g. Cohen's *d*, Pearson's *r*), indicating how they were calculated

Our web collection on statistics for biologists contains articles on many of the points above.

Software and code

Policy information about availability of computer code

Data collection	We employed the following existing software in our data collection: CellRanger (v3.0), Spaceranger (v3.0), Vslide (v1.0.0), bcl2fastq2 (v2.20), ST Pipeline (1.7.6), STAR, HTseq-count (0.7.1), TagGD (0.3.6)
Data analysis	We employed cSplotch (v.0.0.1), a custom code base for Bayesian analysis of ST/snRNA-seq data, in our analysis (https://github.com/adaly/cSplotch). We additionally employed the following existing software: Harmony (0.1.4), SpoTteR (0.0.1), Ilastik (1.4.1), scikit-image (0.18.1), GSEApy (1.1.3), scanpy (1.11.1), Phenograph (1.5.3)

For manuscripts utilizing custom algorithms or software that are central to the research but not yet described in published literature, software must be made available to editors and reviewers. We strongly encourage code deposition in a community repository (e.g. GitHub). See the Nature Portfolio guidelines for submitting code & software for further information.

Data

Policy information about availability of data

All manuscripts must include a data availability statement. This statement should provide the following information, where applicable:

- Accession codes, unique identifiers, or web links for publicly available datasets
- A description of any restrictions on data availability
- For clinical datasets or third party data, please ensure that the statement adheres to our policy

All transcriptomic data used in this study are available on GEO under accession numbers GSE284137 (spatial transcriptomics) and GSE285985 (snRNA-seq)

Research involving human participants, their data, or biological material

Policy information about studies with human participants or human data. See also policy information about sex, gender (identity/presentation), and sexual orientation and race, ethnicity and racism.

Reporting on sex and gender	n/a
Reporting on race, ethnicity, or other socially relevant groupings	n/a
Population characteristics	n/a
Recruitment	n/a
Ethics oversight	n/a

Note that full information on the approval of the study protocol must also be provided in the manuscript.

Field-specific reporting

Please select the one below that is the best fit for your research. If you are not sure, read the appropriate sections before making your selection.

☒ Life sciences ☐ Behavioural & social sciences ☐ Ecological, evolutionary & environmental sciences

For a reference copy of the document with all sections, see nature.com/documents/nr-reporting-summary-flat.pdf

Life sciences study design

All studies must disclose on these points even when the disclosure is negative.

Sample size	A minimum of five mice were samples per time point with a minimum of 9 tissue replicates per mouse. Minimum numbers of mice and replicates were derived by first over-sampling at a given time point, then incrementally downsampling and comparing the results of Bayesian inference between full and down-sampled posteriors using Kullback-Leibler (KL) Divergence.
Data exclusions	Both ST and snRNA-seq measurements were filtered using pre-established thresholds. ST spots with fewer than 100 UMIs were discarded, as were snRNA-seq nuclei with fewer than 800 UMIs, fewer than 800 unique genes, or more than 30% mitochondrial or ribosomal transcripts.
Replication	Select differential expression findings from modeled spatial transcriptomics (ST) data were successfully reproduced using both quantitative IF imaging and an alternative ST platform (10x Genomics' Visium technology). No attempts at reproduction were unsuccessful.
Randomization	All tissue-level covariates -- age, sex, and colon region from which sample was drawn -- are included sources of variation within our hierarchical Bayesian model.
Blinding	Blinding was unnecessary for this study, as all gene expression measurements were gathered in an objective, automated manner and all covariate groups are clearly defined by age, sex, and physical location.

Reporting for specific materials, systems and methods

We require information from authors about some types of materials, experimental systems and methods used in many studies. Here, indicate whether each material, system or method listed is relevant to your study. If you are not sure if a list item applies to your research, read the appropriate section before selecting a response.

Materials & experimental systems

n/a	Involved in the study	
☐	☒	Antibodies
☒	☐	Eukaryotic cell lines
☒	☐	Palaeontology and archaeology
☐	☒	Animals and other organisms
☒	☐	Clinical data
☒	☐	Dual use research of concern
☒	☐	Plants

Methods

n/a	Involved in the study	
☒	☐	ChIP-seq
☒	☐	Flow cytometry
☒	☐	MRI-based neuroimaging

Antibodies

Antibodies used

EPCAM (Biolegend, Alexa-647 labeled primary, clone G8.8, 1:100 dilution), TFF3 (Abcam, unlabeled primary, EPR26048-14, 1:100 dilution), SMA (Abcam, unlabeled primary, clone EPR5368, 1:100 dilution), FABP4 (Abcam, Alexa-647 labeled, clone EPR3579, 1:250 dilution), FABP7 (Abcam, unlabeled primary, clone EPR24033-13, 1:100 dilution), CD48 (Biolegend, APC labeled, clone HM48-1, 1:100 dilution), PRDX6 (Abcam unlabeled primary antibody, clone EPR3754, 1:500 dilution), CD74 (Biolegend unlabeled primary antibody, clone In1/CD74, 1:250 dilution), PDGRFA (Abcam unlabeled primary antibody, clone EPR22059-270, 1:1000 dilution), goat anti-rat IgG (Life Technologies, Alexa Fluor 488 Goat anti-rat IgG, 1:100 dilution), donkey anti-rabbit IgG (Biolegend, Alexa 647 labeled, clone Poly6064, 1:100 dilution)

Validation

All primary antibodies used in this study were validated for specificity and application by the manufacturers, as stated on their respective product datasheets. Where available, validation information was further confirmed by consulting antibody profiles in publicly accessible databases (e.g., UniProt, Human Protein Atlas, CiteAb) and by reviewing published literature citing the same clone in immunofluorescence or immunohistochemistry applications relevant to our tissue type. For each antibody, the clone, host species, labeling, and dilution are specified in the Methods section. In addition to the manufacturer-provided validation, each antibody was tested in-house by performing a serial dilution series on representative tissue sections. Dilutions spanned a range bracketing the manufacturer's recommended concentration; in most cases, dilutions of 1:20, 1:50, 1:100, 1:250, 1:500, and 1:1000 were tested. The optimal dilution was selected based on signal intensity, specificity of staining pattern, and minimal background. This ensured that each antibody produced the expected localization pattern for its target antigen under our staining conditions.
EPCAM (BioLegend, Alexa-647, clone G8.8): Manufacturer validation for flow cytometry and immunofluorescence; documented in Human Protein Atlas with epithelial membrane staining. Literature reports in intestinal epithelia. (Gracz et al. 2010)
TFF3 (Abcam, clone EPR26048-14): Validated by Abcam for IHC and IF, specific staining on goblet cells of mouse colon; literature reports in intestinal and glandular epithelia.
SMA (Abcam, clone EPR5368): Validated by Abcam for IHC/IF in smooth muscle; abundant literature support.
FABP4 (Abcam, Alexa-647, clone EPR3579): Validated for IHC/IF, positive staining in 3T3-L1 cells; documented adipocyte-specific staining in Human Protein Atlas.
FABP7 (Abcam, clone EPR24033-13): Validated for IHC/IF; literature support in astrocytes and glial cells.
CD48 (BioLegend, APC, clone HM48-1): Manufacturer validation for flow cytometry and IF; reported in hematopoietic cells.
PRDX6 (Abcam, clone EPR3754): Validated for IHC/IF; documented cytoplasmic pattern in relevant tissues, including muscle.
CD74 (BioLegend, clone In1/CD74): Manufacturer validation for flow cytometry and IF; literature supports the staining in antigen-presenting cells.
PDGFRA (Abcam, clone EPR22059-270): Validated for IHC/IF; strong literature support for staining mesenchymal cells.
Goat anti-rat IgG (Life Technologies, Alexa Fluor 488) and donkey anti-rabbit IgG (BioLegend, Alexa 647, clone Poly6064): Validated by the manufacturer for immunofluorescent detection of rat or rabbit primaries, respectively.
In all cases, the final working dilution reported in the manuscript reflects the optimal concentration determined from our in-house titration experiments, which balances a high signal-to-noise ratio with the preservation of tissue morphology.

Gracz, Adam D et al. "Sox9 expression marks a subset of CD24-expressing small intestine epithelial stem cells that form organoids in vitro." American journal of physiology. Gastrointestinal and liver physiology vol. 298,5 (2010): G590-600. doi:10.1152/ajpgi.00470.2009

Animals and other research organisms

Policy information about studies involving animals; ARRIVE guidelines recommended for reporting animal research, and Sex and Gender in Research

Laboratory animals

C57BL/6J mice sacrificed at 11 distinct time points: 0w, 1w, 2w, 3w, 4w, 6w, 8w, 12w, 6m, 1yr, and 2yr

Wild animals

n/a

Reporting on sex

Both male and female mice were collected for each age group (31 total male mice, 34 total femals mice across 11 age groups), and sex was accounted for as a covariate within our hierarchical Bayesian model of spatial gene expression. All genes were assessed for sex-driven differential expression at each age group and spatial niche; these results are included in and commented on in our analysis.

Field-collected samples

n/a

Ethics oversight

Mice were maintained in accordance with ethical guidelines monitored by the Institutional Animal Care and Use Committees (IACUC) established by the Division of Comparative Medicine at the Broad Institute of MIT and Harvard and Columbia University, and consistent with the Guide for Care and Use of Laboratory Animals, National Research Council, 1996 (institutional animal welfare assurance no. A4711-01), with protocols 0122-10-16 and AABI3617, respectively.

Note that full information on the approval of the study protocol must also be provided in the manuscript.

Plants

Seed stocks	n/a
Novel plant genotypes	n/a
Authentication	n/a
